# Automatic detection of neuromelanin and iron in the midbrain nuclei using a magnetic resonance imaging‐based brain template

**DOI:** 10.1002/hbm.25770

**Published:** 2022-01-24

**Authors:** Zhijia Jin, Ying Wang, Mojtaba Jokar, Yan Li, Zenghui Cheng, Yu Liu, Rongbiao Tang, Xiaofeng Shi, Youmin Zhang, Jihua Min, Fangtao Liu, Naying He, Fuhua Yan, Ewart Mark Haacke

**Affiliations:** ^1^ Department of Radiology, Ruijin Hospital Shanghai Jiao Tong University School of Medicine Shanghai China; ^2^ SpinTech MRI, Inc. Detroit Michigan USA; ^3^ Department of Radiology Wayne State University Detroit Michigan USA; ^4^ Department of Biomedical Engineering Wayne State University Detroit Michigan USA; ^5^ Department of Neurology Wayne State University Detroit Michigan USA

**Keywords:** automatic boundary detection, brain template models, midbrain nuclei, neuromelanin imaging, quantitative susceptibility mapping

## Abstract

Parkinson disease (PD) is a chronic progressive neurodegenerative disorder characterized pathologically by early loss of neuromelanin (NM) in the substantia nigra pars compacta (SNpc) and increased iron deposition in the substantia nigra (SN). Degeneration of the SN presents as a 50 to 70% loss of pigmented neurons in the ventral lateral tier of the SNpc at the onset of symptoms. Also, using magnetic resonance imaging (MRI), iron deposition and volume changes of the red nucleus (RN), and subthalamic nucleus (STN) have been reported to be associated with disease status and rate of progression. Further, the STN serves as an important target for deep brain stimulation treatment in advanced PD patients. Therefore, an accurate in‐vivo delineation of the SN, its subregions and other midbrain structures such as the RN and STN could be useful to better study iron and NM changes in PD. Our goal was to use an MRI template to create an automatic midbrain deep gray matter nuclei segmentation approach based on iron and NM contrast derived from a single, multiecho magnetization transfer contrast gradient echo (MTC‐GRE) imaging sequence. The short echo TE = 7.5 ms data from a 3D MTC‐GRE sequence was used to find the NM‐rich region, while the second echo TE = 15 ms was used to calculate the quantitative susceptibility map for 87 healthy subjects (mean age ± *SD*: 63.4 ± 6.2 years old, range: 45–81 years). From these data, we created both NM and iron templates and calculated the boundaries of each midbrain nucleus in template space, mapped these boundaries back to the original space and then fine‐tuned the boundaries in the original space using a dynamic programming algorithm to match the details of each individual's NM and iron features. A dual mapping approach was used to improve the performance of the morphological mapping of the midbrain of any given individual to the template space. A threshold approach was used in the NM‐rich region and susceptibility maps to optimize the DICE similarity coefficients and the volume ratios. The results for the NM of the SN as well as the iron containing SN, STN, and RN all indicate a strong agreement with manually drawn structures. The DICE similarity coefficients and volume ratios for these structures were 0.85, 0.87, 0.75, and 0.92 and 0.93, 0.95, 0.89, 1.05, respectively, before applying any threshold on the data. Using this fully automatic template‐based deep gray matter mapping approach, it is possible to accurately measure the tissue properties such as volumes, iron content, and NM content of the midbrain nuclei.

## Graphical abstract

Our goal was to use a magnetic resonance imaging (MRI) template to create an automatic midbrain deep gray matter nuclei segmentation approach based on iron and neuromelanin (NM) contrast derived from a single, multiecho magnetization transfer contrast gradient echo (MTC‐GRE) imaging sequence.

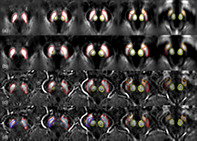

## 1 INTRODUCTION

Parkinson disease (PD) is a chronic progressive neurodegenerative disorder affecting approximately 1% of individuals over 60 years of age (Kowal, Dall, Chakrabarti, Storm, & Jain, 2013). PD is characterized pathologically by early neurodegeneration of neuromelanin (NM) in the substantia nigra pars compacta (SNpc) and increased iron deposition in the substantia nigra (SN) (Dexter et al., 1991; Greenfield & Bosanquet, 1953; He et al., 2021). Degeneration of the SN is a hallmark of the progression of a number of neurodegenerative diseases. In addition to PD, extensive neuronal loss in the SNpc also occurs in atypical parkinsonian disorders including progressive supranuclear palsy (PSP) and multiple system atrophy (MSA), although different subregions of the SN are affected in these disorders (Dexter et al., 1991; Fearnley & Lees, 1991). The SN is composed of two anatomically and functionally distinct regions, the SN pars reticulata (SNpr) and the SNpc. The SNpc contains a dense distribution of NM containing dopaminergic neurons while iron content tends to be higher in the SNpr (Damier, Hirsch, Agid, & Graybiel, 1999; Olszewski & Baxter, 1954). However, clusters of SNpc dopaminergic neurons (known as nigrosomes) are deeply embedded within the SNpr, thus the boundary between the SNpr and the SNpc is difficult to delineate, especially in the caudal region of the SN (Damier et al., 1999). The regional selectivity of PD is relatively specific with a 50 to 70% loss of pigmented neurons in the ventral lateral tier of the SNpc at the onset of symptoms (Cheng, Ulane, & Burke, 2010; Dauer & Przedborski, 2003; Fearnley & Lees, 1991; Ross et al., 2004). In addition to the SN, iron deposition and volume changes of the red nucleus (RN) and subthalamic nucleus (STN) have been reported to be associated with the disease status and rate of progression (Colpan & Slavin, 2010; Lewis et al., 2013). Also, the STN serves as an important target for deep brain stimulation (DBS) treatment in advanced PD patients (DeLong & Wichmann, 2015; Guridi et al., 2018). Inappropriate placement of the DBS electrodes will cause multiple side effects, such as muscle contraction, akinesias, dizziness, and mood changes (Boon et al., 2020; Guehl et al., 2006). Precise preoperative imaging is mandatory in surgical planning to maximize therapeutic benefits and minimize side effects (Lang et al., 2006; T. Liu et al., 2013). Therefore, an accurate and comprehensive in‐vivo delineation of the SN and its subregions, as well as the RN and the STN, could be useful to fully investigate the iron and NM changes in PD and other movement disorders affecting the midbrain.

To date, many studies still use manual or semi‐automated approaches to demarcate the deep brain gray matter (Chen et al., 2014; Sun et al., 2020; Xiong et al., 2020). However, manual segmentation is time‐consuming, especially when large amounts of data need to be evaluated. And, unless the raters are well trained, manual drawings are less reliably duplicated from individual to individual or site to site. Some studies include the use of templates to map iron and/or NM content (Huddleston et al., 2017; Langley et al., 2019; Langley, Huddleston, Sedlacik, Boelmans, & Hu, 2017; Uchida et al., 2020). Creating standardized templates may have a significant impact on recognizing changes in the distribution of iron and NM; automatically calculating the deep gray matter volumes; quantifying the iron content and changes in the NM signal; and, finally, on the reliability of these measurements. Anatomical templates of the SN using conventional magnetic resonance imaging (MRI) sequences have been used previously (Murty et al., 2014; Pauli, Nili, & Tyszka, 2018). Pauli et al. employed T1W and T2W images to create a high‐resolution probabilistic in vivo subcortical nuclei atlas and succeeded in segmenting the SN into the SNpc and SNpr. However, this atlas was based on the images from a young adult population (mean ± SD age: 28.9 ± 3.6 years) which may not be suitable for studies on elderly subjects. Furthermore, both T1W and T2W contrast images cannot be easily used to delineate the highly inter‐digitated boundaries between the SNpc and SNpr in elderly subjects. Based on the anatomical connection of the SN subregions to different parts of the brain (Beckstead, Domesick, & Nauta, 1979; Parent & Hazrati, 1994), several studies used diffusion‐based tractography to segment the SN into the SNpc and SNpr (Menke, Jbabdi, Miller, Matthews, & Zarei, 2010) or subdivide the SN/ventral tegmental area (VTA) into dorsomedial and ventrolateral subregions (Chowdhury, Lambert, Dolan, & Duzel, 2013). However, the estimated structural connectivity has well‐known biases and is highly dependent upon the data acquisition and fiber tracking algorithm and tends to have low resolution (Maier‐Hein et al., 2017; Thomas et al., 2014). Thus, direct visualization and segmentation of the SN and its subregions using high resolution imaging would appear to be a more desirable option especially when it comes to detecting subtle pathological changes of the SN.

The best approach to determine the boundaries of the midbrain nuclei and study pathological changes in PD patients appears to come from the use of T2*‐weighted gradient echo (GRE) imaging and/or susceptibility weighted imaging (SWI) where iron containing regions appear hypointense (Cho et al., 2011; Kwon et al., 2012). The development of quantitative susceptibility mapping (QSM) (de Rochefort et al., 2010; Haacke et al., 2015) enables the quantification of iron stored in ferritin and hemosiderin (Bilgic, Pfefferbaum, Rohlfing, Sullivan, & Adalsteinsson, 2012). Previous studies using QSM have shown that the tissue magnetic susceptibility correlated well with brain iron in PD patients (Acosta‐Cabronero et al., 2017; Ghassaban et al., 2019; Haacke et al., 2015; He et al., 2020). In addition, QSM has been shown to be superior to traditional T2W imaging in preoperative target guidance of DBS (Dimov, Gupta, Kopell, & Wang, 2018). However, QSM alone is not able to separate the SNpc from the SN because both the SNpc and SNpr contain iron. This limitation can be solved by using NM‐sensitive MRI (NM‐MRI) developed in the last few years (Pavese & Tai, 2018). The SNpc and ventral tegmental area (VTA) are mainly composed of the dopaminergic neurons containing NM, but the SNpr is not. Hence, the hyperintense signal seen in NM‐MRI in the midbrain is spatially associated with the SNpc and VTA region as validated by postmortem histological studies (Keren et al., 2015). Thus, the overlap between the NM volume (SNpc plus VTA) and iron containing SN volume (SNpc plus SNpr) is thought to represent the SNpc (He et al., 2021).

So far, there is no study to segment the SNpc accurately and reliably using both QSM and NM‐MRI imaging. Some studies have tried to create an SN atlas using only QSM (Guo et al., 2018; Keuken et al., 2014; Visser, Keuken, Forstmann, & Jenkinson, 2016), while others have created a NM template based on the NM‐MRI imaging using manual drawing, automated segmentation, or artificial intelligence (Ariz et al., 2019; Krupicka et al., 2019; Nakamura, Okada, Kunimatsu, Kasai, & Koike, 2018; Safai et al., 2020). The goal of using a template would be to make the identification of the boundaries easier. However, template mapping is not perfect and the ideal space to mark the boundaries is in the original pristine data space. Whether in template space or original space, simple thresholding methods have their drawbacks. Setting the threshold to higher or lower values can lead to dramatic changes in the estimated volume of the NM or iron content (Safai et al., 2020), especially in PD patients with severe NM degeneration and iron deposition in the SN. Variable contrast in the images can also make it difficult to use a number of algorithms such as region growing. Despite the fact that the SN has high iron content compared to the surrounding regions, it is not uniformly distributed and there are reductions in iron in the nigrosome 1 (N1) territory (Blazejewska et al., 2013). Gaps in the structure such as the N1 territory can also make it difficult to automatically segment the SN and NM regions‐of‐interest (ROIs).

Therefore, in this work, we attempted to create a fully automatic midbrain nuclei segmentation approach based on iron and NM contrast derived from a single, high resolution, multi‐echo magnetization transfer contrast gradient echo (MTC‐GRE) sequence as follows: calculate the boundaries of each structure in template space, map these boundaries back to the original space, and then fine tune the boundaries in the original space using a dynamic programming algorithm (DPA) to match the details of each individual's NM and iron features.

## 2 MATERIALS AND METHODS

### 2.1 Subjects

This study was approved by the local ethics committee and all subjects signed a consent form. Eighty‐seven healthy subjects (mean age ± SD: 63.4 ± 6.2 years old, range: 45–81 years, 53 females) were recruited from the local community by advertisement. The exclusion criteria for the healthy subjects included the following: (a) structural abnormalities, such as tumor, subdural hematoma, or contusion from a previous head trauma; (b) a history of stroke, addiction, neurologic or psychiatric disease, and (c) large‐vessel disease and/or diseases with large volume white matter lesions (i.e., Fazekas grade III).

### 2.2 Data acquisition

MR imaging was carried out on a 3T Ingenia scanner (Philips Healthcare, The Netherlands) using a 15‐channel head array coil. The imaging parameters of the 3D gradient echo SWI sequence with an activated magnetization transfer contrast (MTC) pulse were as follows: shortest TE = 7.5 ms, ΔTE = 7.5 ms with a total of seven echoes, TR = 62 ms, flip angle = 30°, pixel bandwidth = 174 Hz/pixel, matrix size = 384 × 144, slice thickness = 2 mm, slice number = 64, spatial in‐plane resolution = 0.67 × 1.34 mm^2^ interpolated to 0.67 × 0.67 mm^2^, a sense factor of 2, elliptical sampling of k‐space and a total scan time of 4 min 47 s. The MT on‐resonance radio‐frequency pulse used a nominal flip angle of 90°, zero frequency offset, and a set of three‐block pulses each of duration 1.914 ms. The minimum TR was 62 ms because of the specific absorption rate safety considerations. Due to this long repeat time, seven echoes were collected.

### 2.3 Data processing

#### 2.3.1 QSM reconstruction

The first echo of the MTC‐SWI magnitude image (TE = 7.5 ms) was used to delineate the NM content since that provided the key MT contrast. The second echo (TE = 15 ms) was used for QSM reconstruction to evaluate iron deposition in the SN, RN, and STN. The susceptibility maps were created using the following steps: the brain extraction tool, BET (threshold = 0.2, erode = 4, and island = 2000), to segment the brain (Smith, 2002), a 3D phase unwrapping algorithm (3DSRNCP) to unwrap the original phase data (Abdul‐Rahman et al., 2007), sophisticated harmonic artifact reduction (SHARP) to remove unwanted background fields (threshold = 0.05 and deconvolution kernel size = 6) (Schweser, Deistung, Lehr, & Reichenbach, 2011), and a truncated k‐space division (TKD)‐based inverse filtering technique (threshold = 0.1) (Haacke, Tang, Neelavalli, & Cheng, 2010) with an iterative approach (iteration threshold = 0.1 and number of iterations = 4) to reconstruct the final QSM maps (Tang et al., 2013).

#### 2.3.2 Manual ROI segmentation

The ROIs for the NM‐rich region, SN, RN, and STN were manually traced by a single rater on MTC magnitude images and QSM maps zoomed by a factor of four using SPIN software (SpinTech, Inc., Bingham Farms, MI). The NM‐based SN boundaries were traced from the last caudal slice for three to five slices cranially until the NM‐rich region was no longer visible. The iron‐based SN boundaries were traced starting from one slice below the most cranial slice where the STN was visible and continued for four to six consecutive slices to the most caudal slice. The RN ROIs were outlined from the last caudal slice for three to four slices cranially. The STN ROIs were traced from the top of the RN for two slices cranially. For all the ROIs, a DPA was used to determine the final boundaries to alleviate the subjective bias. All these boundaries were then reviewed by a second rater and modified accordingly in consensus with the first rater.

#### 2.3.3 Developing the segmentation approach

##### Preparing the template and creating the atlas

The process for creating the midbrain template used the following steps: the NM template was based on the 7.5 ms echo time MTC data acquisition starting with the original full brain 64‐slice data. One of the best cases was chosen as our template, and the images were zoomed in‐plane by a factor of four for all 64 slices. All other cases were mapped to that template using a global transformation over the central 50 slices. A rigid transformation followed by an affine transformation, and then a b‐spline transformation was applied using the insight segmentation and registration toolkit freeware ITK. The same transformation was used to map the QSM data to the same best case. Practically, we found that these global deformations did not produce good enough template structures in that they were often significantly distorted around the template brain. Therefore, we took that result and cropped the data to 16 slices around the midbrain territory. These volumes were then used to perform a second local template mapping to the best case data. This local deformation transformation significantly improved the template structures. This process was performed using the first 26 cases. The local template results averaged over all 26 cases (the best case and the next 25 cases) had clearer and more consistent boundaries than the global deformation approach. Finally, the data were interpolated in the slice select direction to create a template with 0.167 isotropic resolution (for a total of 192 slices). This was the final template on which the ROIs were drawn.

Continuing the analysis of the template data, the average value served as the probability map for finding the boundaries. To begin the process of defining the structures in the averaged local template, the boundaries for the NM‐rich region, RN, SN, and STN were all drawn manually. For the NM data, the template was drawn on slices 44–98 from the 192 total interpolated slices while for the QSM data slices 44–126 were used. At this point, we ran the DPA for boundary detection to finalize the template boundaries. The DPA used a cost function dependent on the local radius of curvature and signal gradients (Jiang, Dong, & Haacke, 2004; Jiang, Haacke, & Dong, 2007). The full details of this algorithm are given in Data S1, Supporting Information.

##### Determining the original space boundaries from the template boundaries

The next step was to transform the high resolution template boundaries back to the original low resolution space. This required a few preparatory steps. The first step in this process was to find the region to crop in the new dataset. This was done by using a global transformation to the template, and then labeling two central slices of the RN and transforming them back to the original full brain data. The resulting location of the red nucleus in the original image was set to be slice 10 to match the template prior to interpolation in the slice direction (if the RN appeared in two slices, the lower slice was used for slice 10, while if it appeared in 3 slices then the middle slice was used for slice 10). The second step was to take this cropped original space image and zoom it by a factor of four in‐plane and then perform the local deformation of the new case onto the template. Following this, an inverse transform was performed to map the template boundaries back onto the original midbrain data of the new case.

However, there is no guarantee that the boundaries will perfectly match a given individual since every person is different and template mapping is never perfect. Therefore, our goal is to automatically map the template boundaries back onto the individual's structure of interest. To accomplish this we did the following. The third step was to threshold the regions inside the transformed boundary for the QSM data to remove negative values. The fourth step was to find the centerline using image thinning (Zhang & Suen, 1984). The fifth step was to choose an initial starting point for the DPA. Both an Otsu histogram analysis (Otsu, 1979) and a threshold based approach were used to determine if the original boundary extended too far outside the structure of interest. For the NM data, the background intensity was determined from the transformed background ROIs and a constant equal to four times the background SD averaged over all the 25 cases was added to create a threshold below which the signal was set to zero. For the QSM data, the starting threshold was set to zero. When the Otsu threshold yielded a value that caused pixels inside the object of interest to be removed, the template transformed boundary was shrunk accordingly. For the QSM data, if the Otsu threshold was greater than 30 ppb, the threshold was set to 30 ppb. Finally, the resulting boundaries were modified using the same DPA used in the template space. The DPA, along with the RN boundaries, prevents leakage of the SN into the RN and with the help of the template boundaries can differentiate the STN from the SN. It should be noted that all these steps are automatically performed, there is no human intervention.

Given that the VTA also shows NM contrast and abuts the SNpc, and that the separating boundary is not clearly shown in either QSM or NM‐MRI, we obtained the anterior SNpc boundary by subtracting the VTA from the NM to obtain the overlap region (SNpc). This was also done on the manual drawings to provide a fair comparison. The VTA shape appears as a Y as shown in Figure S1.

In summary, boundaries were initially drawn manually in the template space for each of the SN, RN, and STN and the DPA was run to fine tune the boundaries in the template space. These boundaries were then mapped back to the original space where DPA was run once again to provide the final boundaries, making this a fully automated process. From these boundaries, the volumes, signal intensities, and susceptibility values were calculated.

##### Evaluation of the template performance and the DPA process

A total of 87 healthy controls (HCs) were scanned. The SN, STN, and RN were manually traced for all 87 cases. Of these, 30 cases (testing dataset: age ranges 66 ± 7.2 years old, including 17 males and 13 females) were used for the initial testing of the template approach described above. Once all aspects of the algorithm were in place, we then validated the method with the next 57 cases (validation dataset: age ranges 61.9 ± 5.0 years old, including 17 males and 40 females). In order to evaluate the performance of the template, we used two measures: the DICE similarity coefficient, which shows the spatial overlap between the structures associated with the manual and template segmentation methods, and the volume ratio (VR) of the structure determined from the template volume divided by the volume from the manual segmentation.

All data were combined to produce the quantitative information regarding structural volumes (Figure S2) and iron content. The total iron content was calculated by summing the product of the volume and the mean susceptibility of the structure over all the slices that the structure was drawn on. Similarly, the total NM content was calculated from the sum of the product of NM volume and NM contrast over the corresponding slices (Figure S3).

## 3 RESULTS

For the imaging measures discussed in this work, no significant differences between the left and right hemispheres were observed; therefore, all the measurements were averaged between the two hemispheres.

### 3.1 Illustration of the template

**FIGURE 1:**
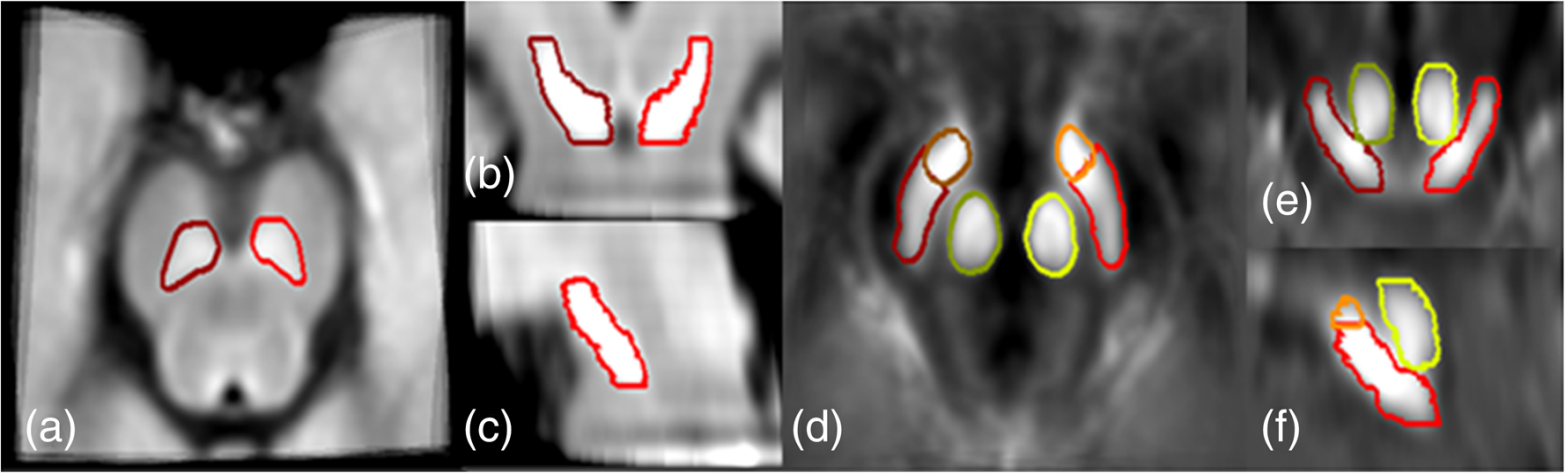
(a–c) Three dimensional perspectives of the NM‐SN from the NM‐MRI template space and (d–f) SN, RN, and STN from the QSM template space. The three orientations are transverse (a, d), coronal (b, e), and sagittal (c, f). Red boundaries: SN; green boundaries: RN; and orange boundaries: STN

**FIGURE 2:**
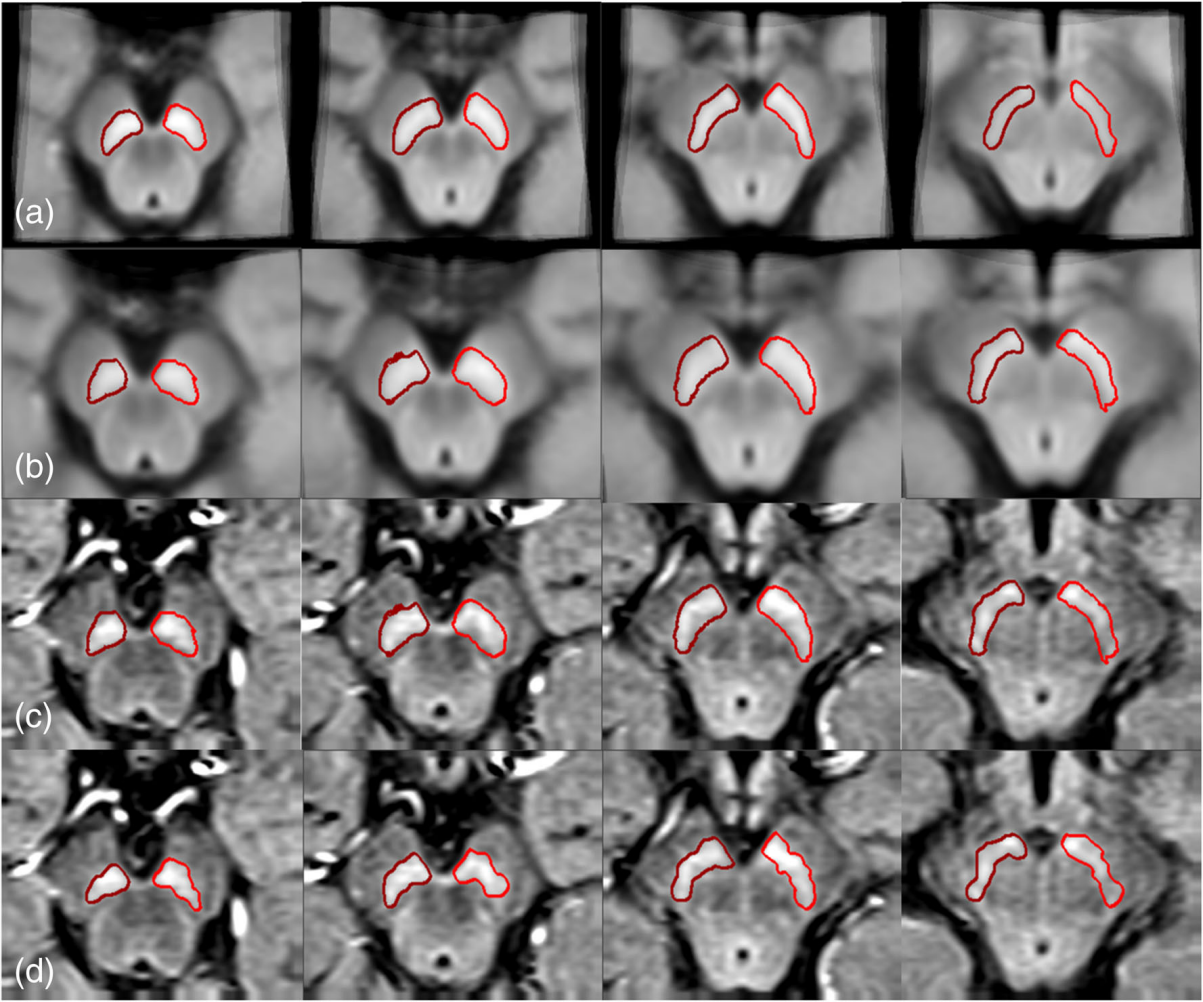
Mapping the boundaries to the original space for the NM‐rich region. Each column represents a different slice. The left most column represents the first most caudal slice where the NM is visualized. Each subsequent slice continues more cranially. (a) Neuromelanin template; (b) the transformed neuromelanin template into the original space; (c) the same boundaries superimposed on the original midbrain images; and (d) the final boundaries after the DPA was applied to get the best fit. Red boundaries: NM

**FIGURE 3:**
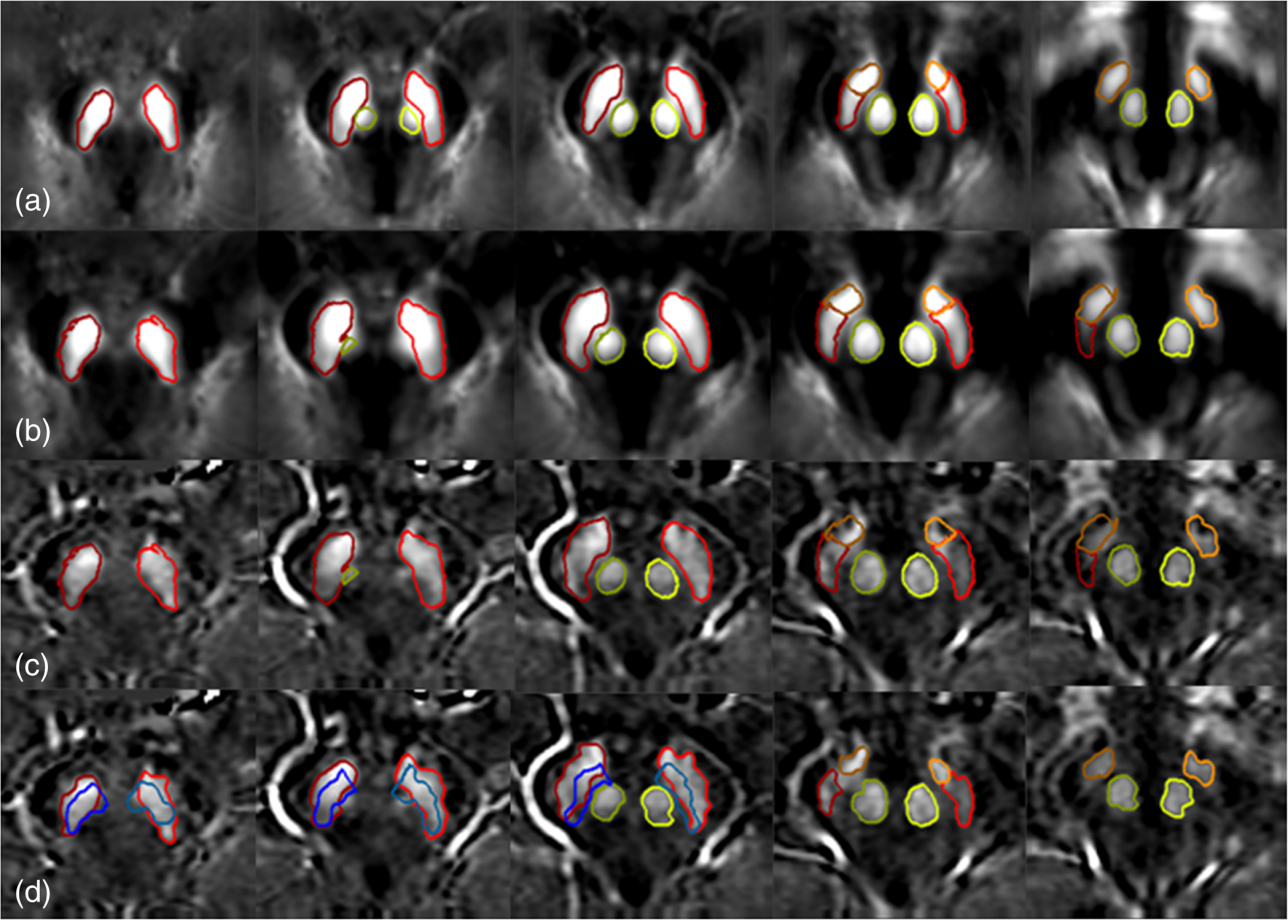
Mapping the boundaries to the original space for the QSM data. Each column represents a different slice. The left most column represents the first most caudal slice where the NM‐rich region is visualized. Each subsequent slice continues more cranially. (a) The QSM template; (b) the transformed QSM template to the original space; (c) the same boundaries superimposed on the original midbrain images; and (d) final boundaries after the DPA was processed to get the best local fit. The SN boundary is shown in red, the STN boundary in orange, and the RN boundary in light green. The blue (dark blue for the right side and light blue for the left side) boundaries show the NM overlaid on the QSM images

A 3D overview of the NM and QSM data after the template boundaries were drawn is shown in Figure 1. The boundaries from the six slices taken from every 12th slice in the 0.167 mm isotropic template space overlaid onto the original 2 mm thick midbrain slices are shown in Figure 2 for the NM data and Figure 3 for the QSM data.

### 3.2 Neuromelanin background measurements

The integrity of the template automatic MTC background intensity measures relative to the manual drawings is shown in Figure S4. The agreement between the manual and template MTC background measures for the first 30 HCs shows a slope of 0.99 and an *R* ^2^ of 0.53, with a *p*‐value <.001. The background values are key to properly thresholding the NM and iron content signals.

### 3.3 Testing and validation results for DICE similarity coefficients and VR measures

The DICE similarity coefficients and VR measures were found for different thresholds for both MTC and QSM images. The higher the threshold, the tighter the distribution becomes in theory eventually approaching unity on both axes. However, the higher thresholds also cause higher loss in volume. Therefore, there is a tradeoff between satisfactory DICE and volume ratios with volume loss. An average VR larger than one indicates that most of the structures found by the fully automated template/DPA approach tended to be larger than those in the manual/DPA approach. Table 1 summarizes the results associated with the estimated template volumes, VR measures and DICE similarity coefficients for each structure. The mean and SD values are shown for the testing and validation datasets as well as for the merged data including all subjects.

**TABLE 1:** Volume estimates, DICE similarity coefficients and volume ratio (VR) mean and SD values of the NM, SN, RN, and STN in the testing dataset, validation dataset, and the merged data before applying any threshold

	Volume (mm^3^)		VR		DICE	
	Mean	SD	Mean	SD	Mean	SD
NM						
Testing dataset	254.06	25.99	0.85	0.07	0.82	0.04
Validation dataset	253.39	23.40	0.98	0.07	0.87	0.03
Overall	253.80	24.16	0.93	0.10	0.85	0.04
SN						
Testing dataset	477.87	49.79	0.93	0.09	0.86	0.04
Validation dataset	493.12	47.77	0.97	0.09	0.87	0.03
Overall	487.98	48.63	0.95	0.09	0.87	0.03
RN						
Testing dataset	191.91	25.90	1.06	0.05	0.93	0.03
Validation dataset	193.72	32.28	1.05	0.07	0.92	0.03
Overall	193.11	30.09	1.05	0.07	0.92	0.03
STN						
Testing dataset	87.32	24.27	0.95	0.19	0.76	0.11
Validation dataset	80.14	24.15	0.86	0.22	0.74	0.13
Overall	82.61	24.29	0.89	0.22	0.75	0.12

#### 3.3.1 Neuromelanin data

**FIGURE 4:**
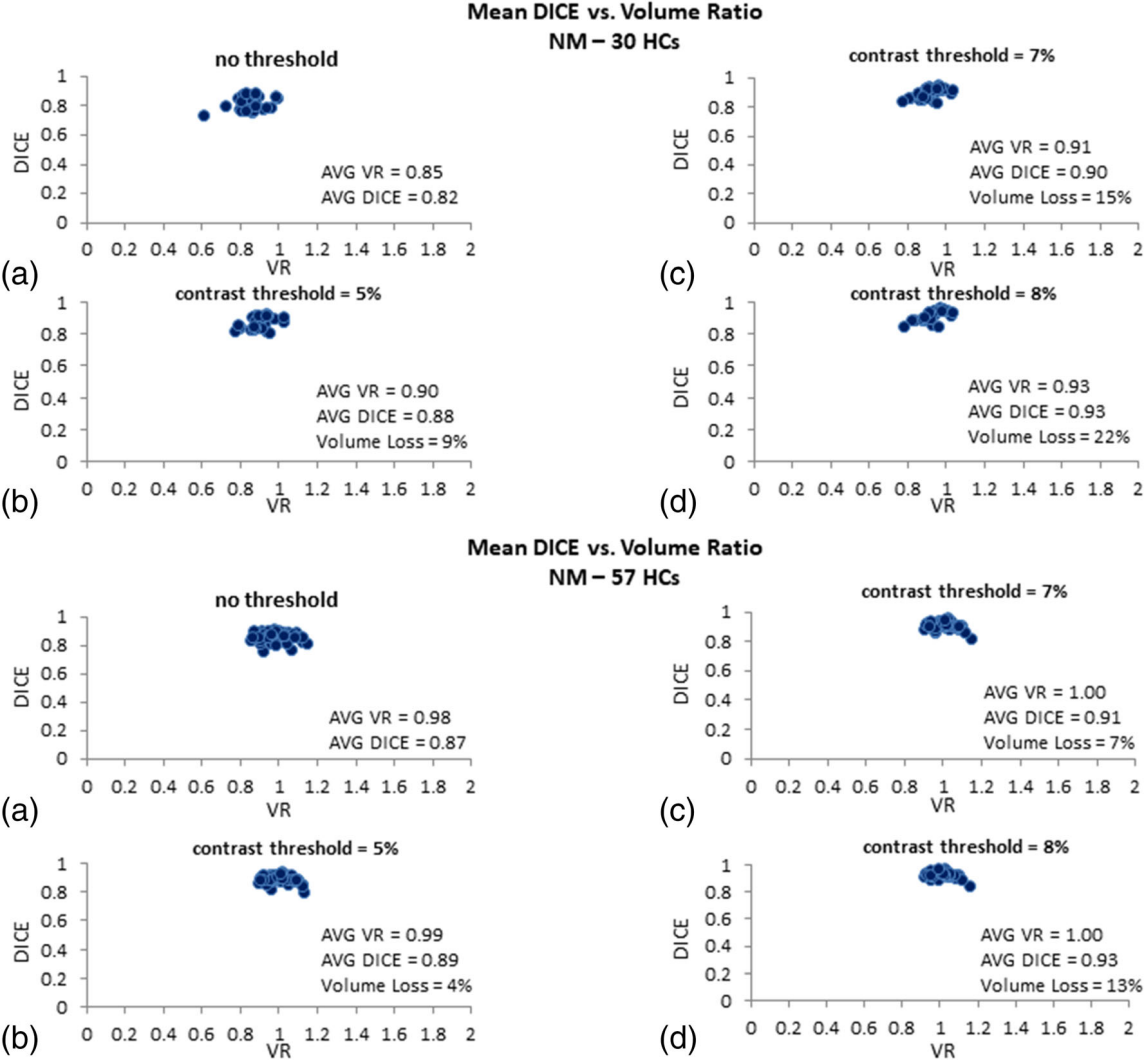
DICE similarity coefficients plotted against volume ratio values for the neuromelanin (NM) based on (a) no threshold, (b) data showing NM contrast larger than 5%, (c) data showing NM contrast larger than 7%, and (d) data showing NM contrast larger than 8% for the 30 test cases (upper plot) and the 57 validation cases (lower plot). The average DICE, volume ratio, and volume loss associated with the template and manually drawn data are quoted for each threshold. A contrast threshold of 5% units keeps the volume loss minimal and yields excellent results

The NM DICE coefficients plotted against the VR for both testing and validation data are shown in Figure 4. For the NM contrast in the testing data, with a threshold of 5% (normalized by the background mean intensity), the average volume loss of the template data for all the cases was less than 10% yielding average DICE and VR values of 0.88 and 0.90, respectively. Higher thresholds such as 7%, yielded an average volume loss slightly over 10% (average DICE = 0.90; average VR = 0.91), and the data showing NM contrast of over 8% yielded an average volume loss of 22% with relatively higher DICE and VR than the lower thresholds (0.93 and 0.93, respectively). The threshold of 5% is seen to keep the volume loss below 10% while resulting in acceptable DICE and VR values. In the validation dataset, with an NM contrast threshold of 5%, the average volume loss of the template data for all the cases was less than 5% yielding an average DICE and VR values of 0.89 and 0.99, respectively.

#### 3.3.2 SN data

**FIGURE 5:**
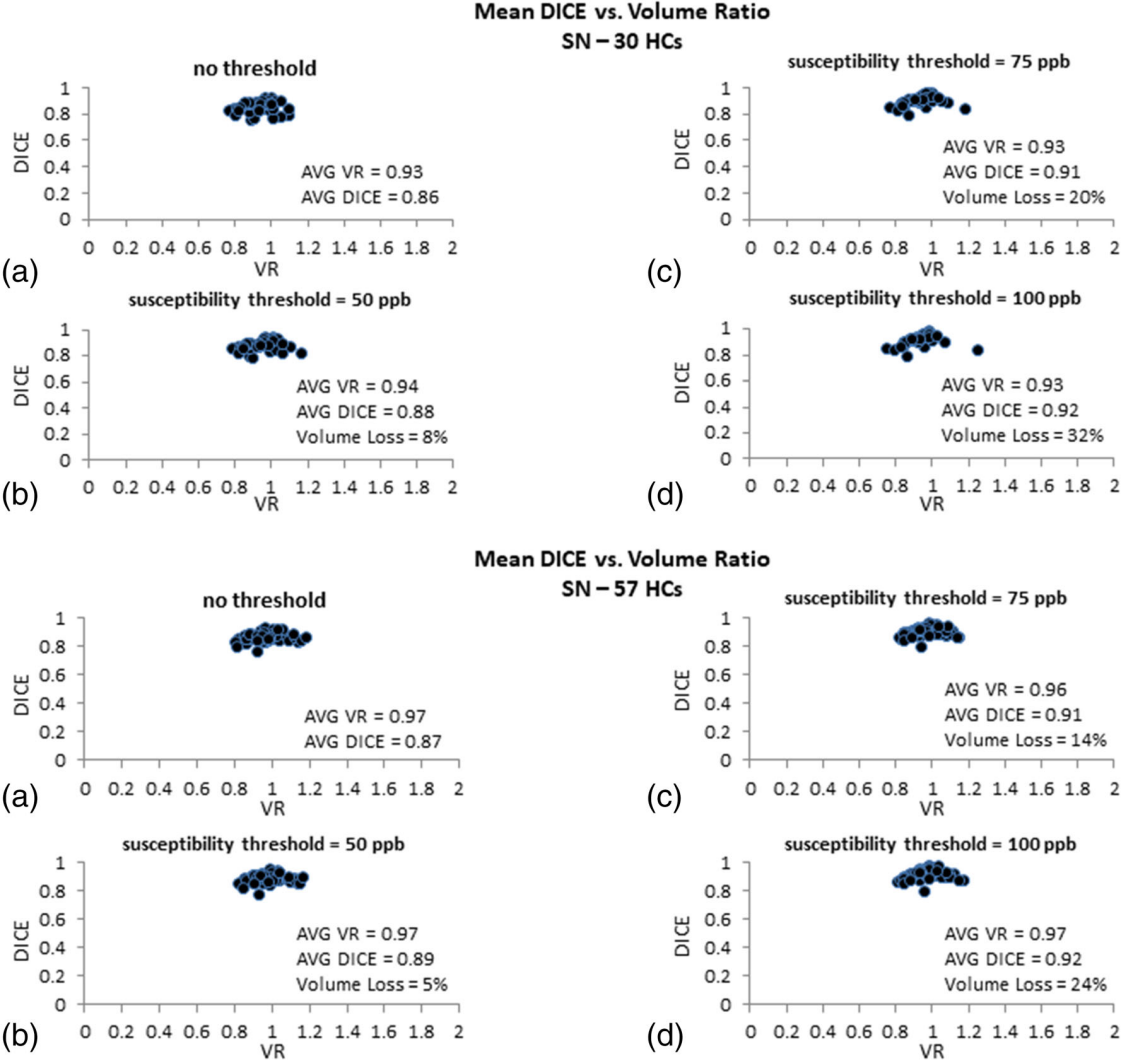
DICE similarity coefficients plotted against volume ratio values for the SN susceptibility based representation: (a) no threshold; (b) data with susceptibility values larger than 50 ppb; (c) data with susceptibility values larger than 75 ppb; and (d) data with susceptibility values larger than 100 ppb for the 30 test cases (upper plot) and the 57 validation cases (lower plot). The average DICE, volume ratio, and volume loss associated with the template and manually drawn data are quoted for each threshold. A threshold of 50 ppb yields excellent results with minimal loss in the SN volume

Similarly, for the iron‐containing SN in the testing data (Figure 5), a mean susceptibility threshold of 50 ppb yielded an average volume loss just under 10% while the average DICE and VR values were 0.88 and 0.94, respectively. In the validation dataset, a threshold of 50 ppb yielded an average volume loss of almost 5% while the average DICE and VR values were 0.89 and 0.97, respectively.

#### 3.3.3 RN data

**FIGURE 6:**
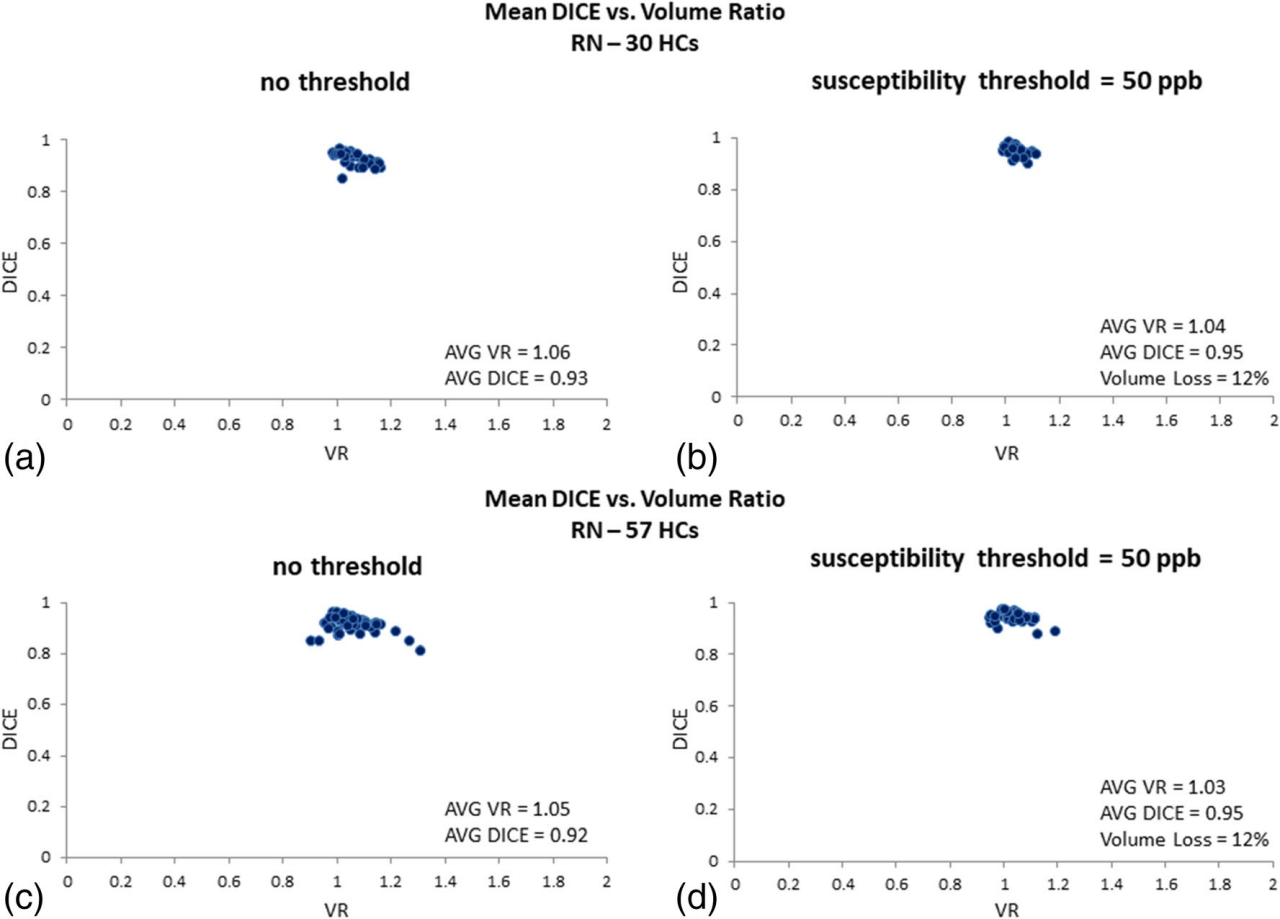
DICE similarity coefficients plotted against volume ratio values for the RN susceptibility based representation: (a, c) before applying any threshold; and (b, d) for a threshold of 50 ppb. The average DICE, volume ratio, and the volume loss associated with the template and manually drawn data for the selected threshold are quoted within the figure. The threshold of 50 ppb limits volume loss to roughly 10%. The data are based on 30 healthy controls from the test dataset and 57 HCs from the validation dataset

Figures 6 illustrates the DICE similarity coefficient versus VR for the RN in both testing and validation datasets. Before applying any threshold on the QSM data, the average DICE and volume ratio of the RN were 0.93 and 1.06, respectively. However, applying a threshold of 50 ppb on the QSM data yielded an average DICE and VR of 0.95 and 1.04, respectively. For the validation dataset, before applying any threshold on the QSM data, the average DICE and VR values of the RN were 0.92 and 1.05, respectively. Using a threshold of 50 ppb on the QSM data yielded an average DICE and VR of 0.95 and 1.03, respectively, and an average volume loss of around 12%.

#### 3.3.4 STN data

**FIGURE 7:**
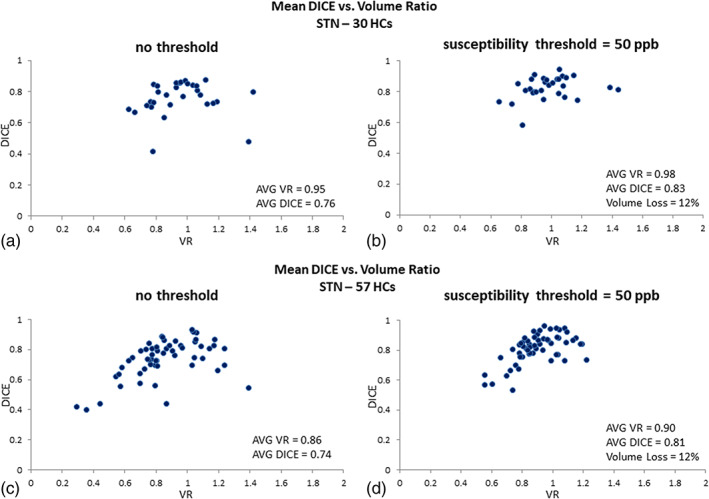
DICE similarity coefficients plotted against volume ratio for the STN susceptibility based representation: (a, c) before applying any threshold; and (b, d) for a threshold of 50 ppb. The average DICE, volume ratio, and the volume loss associated with the template and manually drawn data for the selected threshold are quoted within the figure itself. The 50 ppb threshold keeps the volume loss to about 10%. The data are based on 30 healthy controls for the test dataset and 57 HCs for the validation dataset

The plots of DICE similarity coefficient versus VR for the STN are shown in Figure 7. In the testing dataset, before applying any threshold on the QSM data, the average DICE and volume ratio of the STN were 0.76 and 0.95, respectively. However, applying a threshold of 50 ppb on the QSM data yielded an average DICE and VR of 0.83 and 0.98, respectively. For the validation dataset, before applying any threshold on the QSM data, the average DICE and VR values were 0.74 and 0.86, respectively. Using a threshold of 50 ppb on the QSM data yielded an average DICE and VR of 0.81 and 0.90, respectively, and a template average volume loss of around 12%. There was a much larger spread in the DICE and VR for the STN compared to the other structures.

#### 3.3.5 Overall iron content results

**FIGURE 8:**
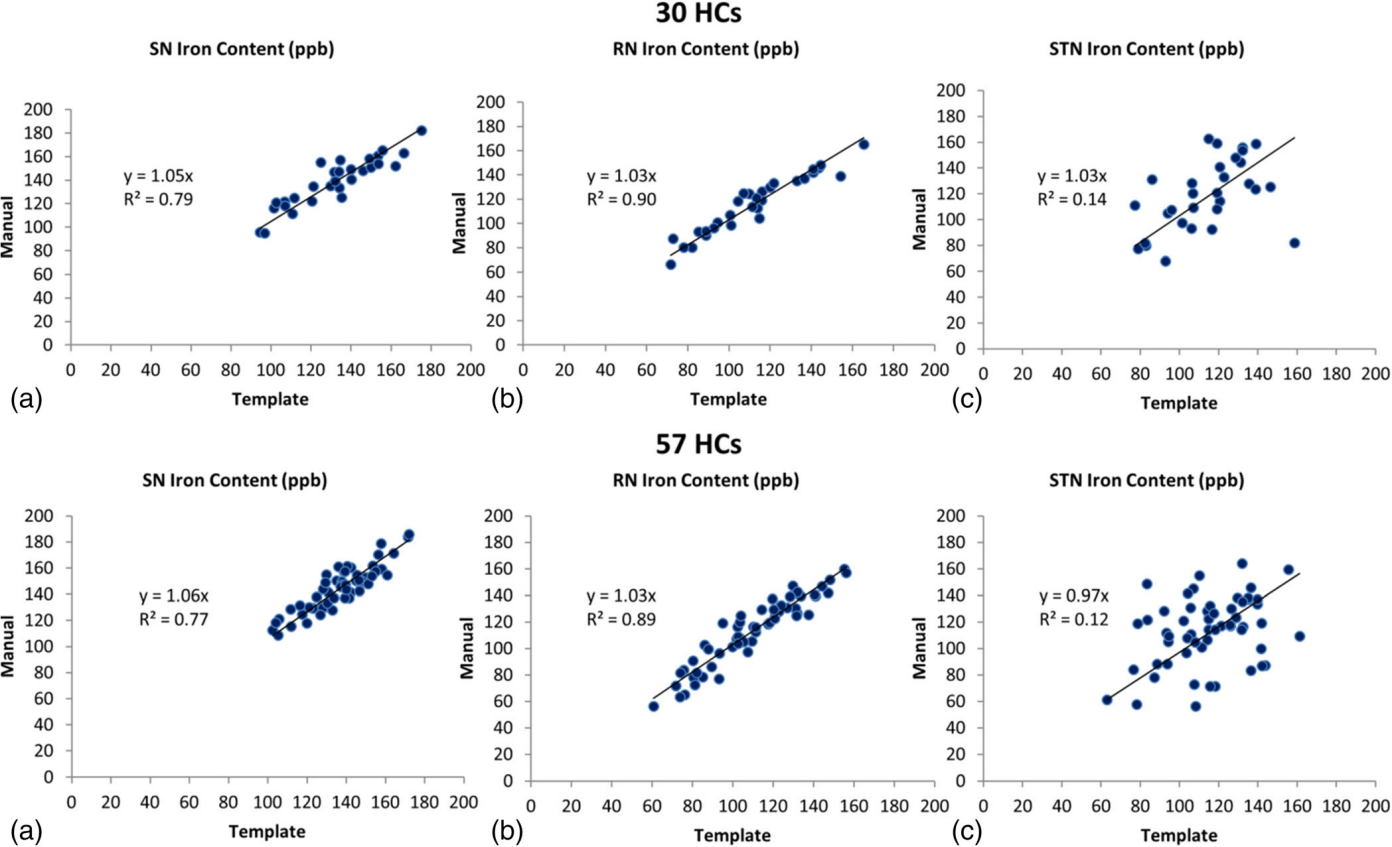
Correlation between the SN, RN, and STN iron content resulting from the manual and template segmentations for the 30 test cases (upper plot)and 57 validation cases (lower plot)

Using a threshold of 50 ppb, the slope and *R* ^2^ values for all three structures, the SN, RN, and STN, are shown in Figure 8. In the testing dataset, the *p*‐values are <.001 for the SN and RN and equal to 0.006 for the STN. In the validation dataset, the *p*‐values are <.001 for the SN and RN and the *p*‐value was equal to .008 for the STN.

## 4 DISCUSSION

In this study, NM and QSM images derived from a single sequence (in less than 5 min) were used for auto‐segmentation of the SN, STN, and RN in the original space, with no need to manually draw ROIs. We described and validated a multicontrast atlas in combination with a DPA for boundary detection in both the template space and in the original images after transforming them back from the template space. Both DICE values and volume ratios as well as the iron content measures, showed excellent agreement between the automatic template approach versus the manual drawings.

### 4.1 Improved morphological mapping and template definition

Practically, we found that using the whole brain global deformable registration with 0.67 mm isotropic resolution, using either ANTs or SpinITK, did not produce morphological transformations matching the shape of the midbrain structures consistently across the subjects. Therefore, we added a local registration step to solve this problem and obtained much improved results. From this local transformation approach, we created both iron and NM templates from a single multiecho sequence. In terms of the validation stage, the second dataset shows that the DICE similarity coefficient and VR values are very close to those from the first dataset for each structure; this confirms the consistency of the results generated by the automated template processing approach proposed herein. Also, the fact that both the DICE and VR values are close to unity shows the excellent performance of this approach. Most public templates do not include the NM‐rich region, SN, STN, and RN. In this work, we interpolated the data first to 0.67 mm isotropic data and then, to allow for refined drawing of the structures (given that they are morphed into new locations after the deformable transformation), they were further interpolated to 0.167 mm isotropic resolution where the template boundaries for each structure were defined.

### 4.2 Age‐related issues in using template mapping

In designing a template for extracting the midbrain structures, age also plays a key role in affecting the segmentation accuracy. The use of an atlas based on only younger healthy subjects may not be appropriate for studying patients with neurodegenerative diseases that affect the structure of the midbrain. These between‐subject and within‐subject age‐ and disease‐related morphometric changes may be important and affect the success of using a template approach on older individuals when localizing the midbrain nuclei. The most appropriate atlas for a given study is the one which requires the least amount of global or regional warping from native subject space to atlas space; therefore, we used elderly HC subjects (mean age: 63.4 ± 6.2 years old) to create the atlas to study patients with neurodegenerative diseases. To alleviate the bias caused by inter‐subject variability, some studies used a probabilistic atlas. Safai et al. (2020) attempted to construct a probabilistic atlas of SNpc based on the NM‐MRI sequence, and then used this atlas to investigate the micro‐structural abnormalities in the SNpc using diffusion MRI in PD patients. They applied a symmetric diffeomorphic registration for registering T1 images and the SNpc masks of 27 HCs onto the MNI space and the atlas was thresholded at 50% probability. However, the average DICE coefficient for the atlas versus human raters was less than 0.61. The thresholding values can lead to dramatic changes in the delineation of the NM, thus decreasing the reproducibility of the atlas. As described above, we used a local deformation to improve the midbrain mapping to the template, and then we mapped the boundary back to the original space and applied the DPA approach to further improve the NM atlas, potentially making it less age dependent and less interpersonal variation dependent.

### 4.3 Comparison to other templates

To our knowledge, no study has used both NM‐MRI and QSM to auto‐segment the SN. Previous research attempted to segment the SN by using structural MRI atlases based on T1‐ and/or T2‐weighted sequences (Dong et al., 2016; Pauli et al., 2018; Plassard et al., 2019). However, the SN is a small structure in the midbrain with low contrast in these images, making it difficult to accurately define the SN boundaries. To overcome this limitation, other studies tried to create the SN atlas using either the NM‐MRI or QSM data (Guo et al., 2018; Keuken et al., 2014; Safai et al., 2020; Visser et al., 2016; Zupan, Suput, Pirtosek, & Vovk, 2019). By using a dynamic atlas composed of NM‐enhanced brain images for the automatic segmentation of the SN, one group showed a DICE value of less than 0.75 (Ariz et al., 2019). The NM‐sensitive T1‐weighted fast spin‐echo sequence applied in their study was not optimal to clearly delineate the SN. The contrast of the NM can be much improved using the MT‐MRI acquisition sequence we describe herein (Y. Liu et al., 2020). Our previous study suggested that using a semi‐automatic approach, the SN mean susceptibility values were (132.12 ± 4.01) ppb averaged over the right and left hemispheres for the healthy controls. In this work, the mean susceptibility of the SN was found to be (124.67 ± 2.31) ppb after averaging over all cases (*p*‐value >.05) (He et al., 2021).

### 4.4 Defining the SNpc

Since QSM provides a quantitative iron measurement in the entire SN (SNpc plus SNpr), and the hyperintense signal seen in NM‐MRI is spatially associated with the SNpc and VTA (Keren et al., 2015), the overlap region of the SN from these two modalities should represent the SNpc. Thus, previous studies using QSM or NM alone may not accurately or reliably define the SNpc. It is well known that iron content is expected to increase in the SNpc while NM is expected to decrease in PD patients. Our auto‐segmentation approach using both NM and QSM in a single sequence is able to reliably separate SNpc and SNpr, and can be used for future studies of the PD and other neurodegenerative disorders.

### 4.5 Mapping the STN

As an important modulator of basal ganglia output, the STN is regularly used as a surgical target of DBS for advanced PD (Dimov et al., 2019; Hamani, Saint‐Cyr, Fraser, Kaplitt, & Lozano, 2004; Iorio‐Morin, Fomenko, & Kalia, 2020). The dorsolateral part of the STN involved in the sensorimotor circuits has been identified as an optimal target of DBS treatment in PD patients (Herzog et al., 2004). However, the STN is difficult to image because of its small size, oblique orientation in three dimensions, and its close proximity to the SN (Ashkan et al., 2007). Milchenko et al. (2018) generated the STN atlas representing normal elder individuals by using T1W and T2W data collected from high‐resolution 7 T MRI. However, the contrast of T1W and T2W is not optimal to accurately delineate the STN. It has been shown that QSM images yield a superior contrast in the depiction of the STN when compared with T2w, T2*w, R2*, phase, and SWI (Chandran, Bynevelt, & Lind, 2016; Dimov et al., 2018; T. Liu et al., 2013). Keuken et al. (2014) created probabilistic maps of the STN based on manually segmented multimodality data (including T1W, T2*‐weighted and QSM) of 30 young healthy participants scanned on a 7 T system. However, their probabilistic maps are based on data from young healthy participants, which may not be appropriate for older people given potential age‐related morphological brain changes, such as an increase in ventricle size and the displacement of subcortical nuclei (Fjell & Walhovd, 2010; Keuken et al., 2013). As discussed earlier, this limitation can be overcome by delineating the STN in the individual's original space based on the high resolution QSM data in our study.

### 4.6 Limitations

There are, however, some limitations to this study. First, the template was only tested for a single resolution. This should not be a problem for higher resolution datasets, since we created a very high‐resolution set of template boundaries for the different structure. The background signal will vary from site to site perhaps and most assuredly from manufacturer to manufacturer. However, the automated background regions should alleviate this problem, although the average SD will need to be carefully determined. The thresholds for the NM were defined in terms of a percentage of the contrast with respect to the background signal, making it less dependent on manufacturer's scaling methods. Second, only those protocols collecting both QSM and NM can be used if there is interest in isolating the SNpc from the overlap of the NM‐rich region and the SN iron containing boundaries. In our case, a single sequence is used to accomplish this. Third, the STN was difficult to assess because of its overlap with the SN in the anterior connecting region. Possible solutions to this include collecting the data with higher resolution and reformatting the data to ensure that the SN for the most part sits on top of the SN.

In conclusion, our combined global and local transformation approach to template space, along with the three structure template mapping, background determination, and refined boundary detection provide for a robust approach to automating the quantification of NM and iron in the midbrain. Furthermore, the overlap between the iron content of the SN and the NM provides a means to isolate the SNpc. With this approach, it is possible to quantify the NM in the SN and the volume and iron content for all of the SN, STN and RN. This approach should make it possible to study the changes in these imaging biomarkers for a variety of neurodegenerative diseases without the need for manual tracing of these structures. We plan to apply this approach next to a cohort of Parkinson patients to see if the resulting biomarkers of NM volume, SN volume and SN iron content could ultimately distinguish PD patients from healthy controls.

## CONFLICT OF INTEREST

The authors declare no potential conflicts of interest.

## AUTHOR CONTRIBUTIONS

Y.W.: writing—original draft; data curation; investigation; formal analysis; Z.J.: writing—original draft; data curation; investigation; formal analysis. M.J.: writing—original draft; data curation; investigation; formal analysis. Ya.L.: writing—original draft; methodology; formal analysis. Z.C.: writing—review and editing; investigation. YuL.: writing—review and editing; investigation. R.T.: writing—review and editing; investigation. X.S..: writing—review and editing; investigation. Y.Z.: writing—review and editing; investigation. J.M.: data acquisition and investigation. F.L.: data acquisition and investigation. N.H.: conceptualization; supervision; funding acquisition; resources; writing—review and editing. F.Y.: conceptualization; supervision; funding acquisition; resources; writing—review and editing. E.M.H.: conceptualization; supervision; resources; writing—review and editing. project administration.

## Supporting information


**Table S1** NM volume and contrast values for four volunteers who were scanned five times each at four different sites
**Table S2**. SN volume and mean susceptibility values for four volunteers who were scanned five times each at four different sites
**Table S3**. RN volume and mean susceptibility values for four volunteers who were scanned five times each at four different sites
**Table S4**. STN volume and mean susceptibility values for four volunteers who were scanned five times each at four different sites.
**Figure S1**. Four consecutive midbrain NM images. (a, b) The yellow boundary is the VTA structure. (c, d) The VTA is removed from the NM‐rich region shown in the corresponding slices
**Figure S2**. Volume of the NM‐rich region, RN, SN and STN as a function of age associated with the merged dataset (87 healthy controls). The *p*‐values associated with each correlation are 0.30, 0.61, 0.05, and 0.40, respectively
**Figure S3**. **(**a) Total iron content of the SN (*p* = 0.02), and (b) total NM content (*p* = 0.21) as a function of age for the merged dataset (87 healthy controls)
**Figure S4**. Agreement between the NM background manual and template measurements for 30 healthy controls. NM measures are in arbitrary units
**Figure S5**. Correlation between the NM mean intensity measurements resulting from the manual and template segmentations for the (a) 30 test cases, and (b) 57 validation cases. (*p*‐value <0.001 for both plots)
**Figure S6**. (a) NM boundary before DPA, (b) Background boundaries shown relative to the NM boundary after DPA. The orange boundary is the background region after transformation from the template space to the original space. Using the background value plus 1,000 makes it easier to refine the DPA boundary to provide a faster convergence and to use a smaller and safer search radius to prevent the algorithm from leaking outside the original boundary
**Figure S7**. The choice of α can dramatically affect the final DPA boundary. (a) *α* = 0.05; (b) *α* = 0.10; (c) *α* = 0.15; and (d) *α* = 0.20. Note that lower values of *α* lead to less smoothing from the radius constraint, while larger values of *α* lead to more smoothing
**Figure S8**. **(**a) No noise case: the model image shows a rectangular shape with two different intensities. (b) A region was drawn around the central area, (c) and then the centerline was found and updated after applying the DPA for five iterations. (d) With noise: the same drawing was then used in the presence of a CNR of 7:1 based on the difference in the signal intensities of the central region and the framed region within the second rectangular boundary. (e) After five iterations, the correct boundary was found providing the correct area of 1,400 pixels
**Figure S9**. **(**a) No noise case: the model image shows the moon shape with two different intensities. (b) A region was drawn around the central area, (c) and then the centerline was found and updated after each of the DPA for five iterations. (d) First 10 iterations, (e) then 15 iterations, (f) and then with just five iterations using the adaptive Otsu threshold approach were evaluated. (g) With noise: The same drawing was then used in the presence of a CNR of 7:1 based on the difference in the signal intensities of the central region and the framed region within the second rectangular boundary, (h) and again using just five iterations using the adaptive Otsu threshold approach
**Figure S10**. (a) No noise case: the model image shows the cashew shape with two different intensities. (b) A region was drawn around the central area, (c) and then the centerline was found and updated after DPA five iterations. (d) With noise: The same drawing was then used in the presence of a CNR of 7:1 based on the difference in the signal intensities of the central region and the framed region within the second rectangular boundary. (e) The final result
**Figure S11**. Flowchart of the automated segmentation algorithm
**Data S1**. Supporting Information

## Data Availability

The source and means of obtaining the data used in this paper have been described in the Methods section. The codes in this study are available upon a reasonable request to the corresponding author, E. Mark Haacke. The data is available via a reasonable request with a formal data sharing agreement from the corresponding author.
