# Supplementary material for: Automatic detection of neuromelanin and iron in the midbrain nuclei using a magnetic resonance imaging‐based brain template
Source: Hum Brain Mapp. 2022 Jan 24;43(6):2011–25. doi: 10.1002/hbm.25770 (PMC8933249; doi:10.1002/hbm.25770)
Supplement: Supplementary file 1 — Table S1 NM volume and contrast values for four volunteers who were scanned five times each at four different sites Table S2. SN volume and mean susceptibility values for four volunteers who were scanned five times each at four different sites Table S3. RN volume and mean susceptibility values for four volunteers who were scanned five times each at four different sites Table S4. STN volume and mean susceptibility values for four volunteers who were scanned five times each at four different sites. Figure S1. Four consecutive midbrain NM images. (a, b) The yellow boundary is the VTA structure. (c, d) The VTA is removed from the NM‐rich region shown in the corresponding slices Figure S2. Volume of the NM‐rich region, RN, SN and STN as a function of age associated with the merged dataset (87 healthy controls). The p‐values associated with each correlation are 0.30, 0.61, 0.05, and 0.40, respectively Figure S3. (a) Total iron content of the SN (p = 0.02), and (b) total NM content (p = 0.21) as a function of age for the merged dataset (87 healthy controls) Figure S4. Agreement between the NM background manual and template measurements for 30 healthy controls. NM measures are in arbitrary units Figure S5. Correlation between the NM mean intensity measurements resulting from the manual and template segmentations for the (a) 30 test cases, and (b) 57 validation cases. (p‐value <0.001 for both plots) Figure S6. (a) NM boundary before DPA, (b) Background boundaries shown relative to the NM boundary after DPA. The orange boundary is the background region after transformation from the template space to the original space. Using the background value plus 1,000 makes it easier to refine the DPA boundary to provide a faster convergence and to use a smaller and safer search radius to prevent the algorithm from leaking outside the original boundary Figure S7. The choice of α can dramatically affect the final DPA boundary. (a) α = 0.05; (b) α = 0.10; (c) α = 0.15; and (d) α = 0.20. No [file HBM-43-2011-s001.docx]

**Automatic Detection of Neuromelanin and Iron in the Midbrain Nuclei using an MRI Based Brain Template**

Zhijia Jin^1+^, Ying Wang^2,3+^, Mojtaba Jokar^2+^, Yan Li^1^, Zenghui Cheng^1^, Yu Liu^1^, Rongbiao Tang^1^, Xiaofeng Shi^1^, Youmin Zhang^1^, Jihua Min^1^, Fangtao Liu^1^, Naying He^1*^, Fuhua Yan^1*^, E. Mark Haacke^1,2,3,4,5*^

*^1^* *Department of Radiology, Ruijin Hospital, Shanghai Jiao Tong University School of Medicine, Shanghai, China*

*^2^ Magnetic Resonance Innovations, Inc., Bingham Farms, MI, USA*

*^3^* *Department of Radiology, Wayne State University, Detroit, MI, USA*

*^4^ Department of Biomedical Engineering, Wayne State University, Detroit, Michigan, USA*

*^5^ Department of Neurology, Wayne State University, Detroit, MI, USA*

**^+^ These three authors made equal contributions**

**^*^Correspondence to:**

E. Mark Haacke, PhD

Department of Radiology, Wayne State University, Detroit, MI, USA

E-mail: [nmrimaging@gmail.com](mailto:nmrimaging@gmail.com)

Fuhua Yan, MD, PhD

Department of Radiology, Ruijin Hospital, Shanghai Jiao Tong University School of Medicine

No.197 Ruijin Er Road, Shanghai, 200025, China

E-mail: [yfh11655@rjh.com.cn](mailto:yfh11655@rjh.com.cn)

Naying He, MD, PhD

Department of Radiology, Ruijin Hospital, Shanghai Jiao Tong University School of Medicine

No.197 Ruijin Er Road, Shanghai, 200025, China

E-mail: [hny12267@rjh.com.cn](mailto:hny12267@rjh.com.cn)

**Supplementary Material**

**Neuromelanin background measurements**

As discussed in the main manuscript, in order to obtain a more accurate structure, the VTA was removed from the NM boundaries for all the cases, as shown in the **Supplementary Figure 1**. The volumes of the NM-rich region, RN, SN and STN resulting from the template segmentation are shown in **Supplementary Figure 2** for all the cases. There is a significant reduction in volume of the SN with age. **Supplementary Figure 3** illustrates the total SN iron content and the total NM content as a function of age for all the cases. The total NM content was calculated from the sum of the product of NM volume and its contrast over the slices where NM was present and the total iron content was calculated by summing the product of the volume and the mean susceptibility of the structure over all the slices where the SN was present.

Since the background values are key to properly thresholding the NM signals, 30 MTC cases were processed to obtain the NM background measurements. The integrity of the template automatic MTC background intensity measures relative to the manual drawings is shown in the **Supplementary Figure 4**. The agreement between the manual and template MTC background measures shows a slope of 0.99, an R^2^ of 0.53, and a p-value < 0.001.

There is strong agreement between the manual and the template segmentation for the NM mean intensity measurements for both datasets (**Supplementary Figure 5**).

**Description of the Template Boundary Building Process**

The final template boundaries of all the structures of interest were determined using a dynamic programming algorithm (DPA) for boundary detection in both the template space and the original space. The detailed steps of the DPA algorithm are as follows:

1. The initial boundary was drawn on the template data for both NM-rich region and QSM.
2. The background was drawn on the template images.
3. The boundaries and background were then transformed to the original space. (**Supplementary Figure 6**)
4. The background mean + 4σ (where σ is the standard deviation of the background region of interest which in this study was found to be roughly 250 units) was used as the threshold on the MTC data to remove all points lower than this threshold to determine the NM refined boundary. For the QSM data, the pixels with a susceptibility value less than zero ppb were removed before the DPA was run for the SN, STN and RN.
5. A 3×3 Gaussian filter was used to smooth the initial boundary.
6. The centerline was determined using the Zhang-Suen thinning method ([Zhang & Suen, 1984](#_ENREF_4)).
7. *Boundary refinement prior to running DPA:* Since there can be some variation of intensity around the structure, the global threshold might not be good for the whole structure in the MTC data. Therefore, we used Otsu's method ([Otsu, 1979](#_ENREF_3)) on the filtered local image in a 40x40 pixel square around each single centerline point and removed all points lower than the Otsu threshold. If the initial boundary was outside the boundary obtained by the Otsu threshold, the boundary was modified to those nearest points that remain (referred to as the Otsu boundary). This step helps the DPA converge faster.
8. *Implementing DPA:* For each point along the centerline, the DPA was run in the corresponding search box. To allow for curved shapes, the center of the centerline was used to determine the initial rays. Then DPA was applied for the next set of rays by shifting one pixel along the centerline associated with the initial boundary. For each DPA iteration the centerline was updated. Once the endpoint was reached, these rays were swept through 180°. Then the algorithm returns along the centerline until it reaches the opposite endpoint sweeping through another 180°. Finally, the center point moves back along the centerline returning to the starting point to close the boundary. This process was repeated five times searching both inside and outside the boundary for the best result. For the NM region, STN and the SN, this step is followed by five more iterations using only an inside search.
9. VTA removal: Any area from the detected SN overlapping with the VTA boundary was removed from the final estimates of the NM boundary. (**Supplementary Figure 1**)

**Description of the Dynamic Programming Algorithm (DPA)**

The cost function used in this DPA consists of a derivative term as well as a radius of curvature term which avoids the leakage of the structure of interest into the adjacent objects, although we limited the search radius to four pixels both inside and outside the boundary in the zoomed space. ([Jiang, Haacke, & Dong, 2007](#_ENREF_1)) For points outside the boundary, when searching from the centerline outward, negative derivative values were set to zero. This restriction prevented the boundary from leaking out into nearby bright objects. The cost function is given by:

$$C\left( r,m \right)=\frac{G\left( r,m \right)+G_{pre}}{G_{max}}-\alpha*\frac{|R\left( r,m \right)-R_{avg}|}{R_{avg}}$$

where

$$G_{pre}=\sum_{t=m-3}^{m-1} {G\left( t \right)}_{max}$$

and the gradient and the radius along the m^th^ ray at the r^th^ point are denoted by $G\left( r,m \right)$ and $R\left( r,m \right)$, respectively. The term $G_{max}$ represents the maximum derivative inside the image and $R_{avg}$ is the average radius over the previous three radii. The constant α represents the relative weighting of the derivative and radius terms which can be set to values between 0 and 1. The closer the shape of the structure of interest is to a circle the higher α can be set. If there are sharp edges, these will be smoothed by a large choice for α. Values between 0.05 and 0.15 all did a similar job in finding the edges faithfully, so a value of α = 0.1 was chosen to be conservative (**Supplementary Figure 7**). Higher values tended to round out the structure and shrink it more toward a circle. Low values yielded a very sharp edged boundary. Finally, the candidate points for the new boundary were selected by maximizing the above-mentioned cost function for each ray.

Some example simulations are shown for different shapes. In **Supplementary Figures 8-10**, the signal for the central region was set to 100 units, the signal for the outer framed area within the second boundary but outside the first boundary was set to 30 units and the background outside the second boundary was set to zero. Gaussian noise with a mean of zero and a standard deviation of 10 units was added to the images. The first example of a rectangle with sharply defined corners and 1400 pixels is shown in **Supplementary Figure 8**. Despite the presence of noise, the boundary was still found perfectly.

As a more realistic case, a crescent shaped object with 964 pixels inside was chosen to mimic the more difficult case of detecting the boundary for a curved object. **Supplementary Figure 9** shows the effects of running a different number of iterations for this object. The mean after running 30 iterations following the initial 5 iterations was found to be 990.5 with a standard deviation of 1.5 while using the Otsu approach in the presence of an SNR of 10:1, the mean was 984 with a standard deviation of 2.0 (**Supplementary Figure 9f**).

Finally, a cashew shape with 1046 pixels inside was chosen to mimic the SN and evaluate the method when no sharp edges are present. After running 30 iterations following the initial 5 iterations, the mean was found to be 1086.5 with a standard deviation = 2.5 (**Supplementary Figure 10c**). Using the Otsu approach in the presence of a SNR of 10:1, and 35 iterations, the mean was found to be 1097 with a standard deviation of 0.0.

**Supplementary Figure 11** shows the diagram summarizing the steps in the template algorithm.

**Validating the reproducibility of the results obtained by the template algorithm**

In order to further investigate the consistency and reproducibility of the results generated by the template algorithm presented in this work, the neuromelanin and iron content were evaluated for four healthy volunteers. The volunteers were imaged five times each at different sites (subject 1: female, 23 years old; subject 2: male, 23 years old; subject 3: female, 45 years old; subject 4: female, 28 years old) with Siemens PRISMA 3T scanners. None of these volunteers had a personal or family history of PD, or any neurological or mental disease, a history of psychological disease or drug usage or cardiovascular or cerebrovascular disease.

A resolution of 0.67mm x 1mm x 1.34mm interpolated to 0.67mm isotropic resolution in-plane was used for each 3D GRE. This resolution proved whole brain coverage, optimal resolution and SNR, and yet keep the scan time to 5 minutes or less to avoid patient motion. All 3D GRE scans used the same field-of-view, a TR of 29ms, two different flip angles of 6^o^ and 27^o^, and echo times of 7.5ms, 15ms and 22.5ms.. For the MTC scans, 5 echoes were collected starting at 7.5ms with increments of 7.5ms, a TR of 62ms and two flip angles of 12^o^ and 30^o^. The MTC-STAGE scans covered the central part of the brain including the midbrain and locus coeruleus territory. This was accomplished by centering the slab at the top of the fourth ventricle from the sagittal pilot scan. All other scans had full brain coverage. Each manufacturer had its own type of MT pulse. Specifically, this included: an off-resonance pulse for Siemens with a 10ms single-lobe Gaussian pulse, 1,200 Hz off-resonance and a nominal flip angle of 500^o.^ Then, in order to find the final boundaries, the template algorithm was applied as explained in the main manuscript. The product of the simulated MTC data from the flip angles 15 and 40 was used in order to do the template mapping associated with these four cases. ([Liu et al., 2020](#_ENREF_2))

Supplementary Table 1 shows the average and standard deviation of the NM volume and normalized contrast (with respect to the background) over all 5 scans for subjects 1-4. The mean ± standard error for the right/left hemispheres associated with these subjects were calculated as 267 ± 15.3 mm^3^/273 ± 4.2 mm^3^; and 20.1 ± 1.97%/21.8 ± 1.52% for the NM volume and normalized NM contrast, respectively. Supplementary Tables 2-4 show the average and standard deviation of the SN, RN, and STN volumes and mean susceptibility values over all 5 scans for subjects 1-4, respectively. The mean ± standard error of the volume for the right/left hemispheres associated with these subjects were 383 ± 23.7 mm^3^/387 ± 22.4 mm^3^; 142 ± 5.4 mm^3^/151 ± 21.2 mm^3^; and 54 ± 9.1 mm^3^/45 ± 13.1 mm^3^ for the SN, RN and STN, respectively. The mean ± standard error of the mean susceptibility values for the right/left hemispheres associated with these subjects were 154 ± 14.8 ppb/146 ± 16.1 ppb; 72 ± 8.4 ppb/66 ± 10.6 ppb; and 91 ± 7.2 ppb/95 ± 14.8 ppb for the SN, RN and STN, respectively.

| Cases | NM Volume (mm^3^)-  Right Hemisphere | | NM Volume (mm^3^)-  Left Hemisphere | | Normalized NM Mean Contrast-Right Hemisphere | | Normalized NM Mean Contrast-Left Hemisphere | |
| --- | --- | --- | --- | --- | --- | --- | --- | --- |
|  | Mean | Standard Deviation | Mean | Standard Deviation | Mean | Standard Deviation | Mean | Standard Deviation |
| subject 1 | 244 | 22.5 | 205 | 27.3 | 16.1% | 3.17% | 18.9% | 1.53% |
| subject 2 | 300 | 21 | 378 | 43.2 | 21.1% | 2.66% | 21.0% | 1.34% |
| subject 3 | 238 | 18.3 | 236 | 24.2 | 25.1% | 2.79% | 26.1% | 2.62% |
| subject 4 | 286 | 33.3 | 272 | 32.8 | 18.2% | 1.29% | 21.2% | 1.62% |
| Total | Average mean | Standard error of the mean | Average mean | Standard error of the mean | Average mean | Standard error of the mean | Average mean | Standard error of the mean |
|  | 267 | 15.3 | 273 | 4.2 | 20.1% | 1.97% | 21.8% | 1.52% |

**Supplementary Table 1.** NM volume and contrast values for four volunteers who were scanned 5 times each at four different sites.

| Cases | SN Volume (mm^3^)-Right Hemisphere | | SN Volume (mm^3^)-Left Hemisphere | | SN Mean Susceptibility (ppb)-Right Hemisphere | | SN Mean Susceptibility (ppb)-Left Hemisphere | |
| --- | --- | --- | --- | --- | --- | --- | --- | --- |
|  | Mean | Standard Deviation | Mean | Standard Deviation | Mean | Standard Deviation | Mean | Standard Deviation |
| subject 1 | 385 | 6.0 | 360 | 59.8 | 188 | 11.8 | 177 | 11.8 |
| subject 2 | 415 | 29.4 | 439 | 38.2 | 125 | 4.9 | 112 | 6.0 |
| subject 3 | 316 | 43.7 | 340 | 48.7 | 133 | 6.8 | 126 | 7.1 |
| subject 4 | 417 | 30.0 | 408 | 32.1 | 170 | 5.9 | 169 | 5.6 |
| Total | Average mean | Standard error of the mean | Average mean | Standard error of the mean | Average mean | Standard error of the mean | Average mean | Standard error of the mean |
|  | 383 | 23.7 | 387 | 22.4 | 154 | 14.8 | 146 | 16.1 |

**Supplementary Table 2.** SN volume and mean susceptibility values for four volunteers who were scanned 5 times each at four different sites.

| Cases | RN volume (mm3)-Right Hemisphere | | RN volume (mm3)-Left Hemisphere | | RN Mean Susceptibility (ppb)-Right Hemisphere | | RN Mean Susceptibility (ppb)-Left Hemisphere | |
| --- | --- | --- | --- | --- | --- | --- | --- | --- |
|  | Mean | Standard Deviation | Mean | Standard Deviation | Mean | Standard Deviation | Mean | Standard Deviation |
| subject 1 | 150 | 4.4 | 178 | 16.0 | 78 | 8.1 | 68 | 10.5 |
| subject 2 | 129 | 15.2 | 92 | 15.9 | 48 | 1.1 | 41 | 1.9 |
| subject 3 | 138 | 7.1 | 147 | 9.0 | 74 | 5.3 | 62 | 6.7 |
| subject 4 | 152 | 11.9 | 185 | 15.3 | 88 | 9.1 | 92 | 3.8 |
| Total | Average mean | Standard error of the mean | Average mean | Standard error of the mean | Average mean | Standard error of the mean | Average mean | Standard error of the mean |
|  | 142 | 5.4 | 151 | 21.2 | 72 | 8.4 | 66 | 10.6 |

**Supplementary Table 3.** RN volume and mean susceptibility values for four volunteers who were scanned 5 times each at four different sites.

| Cases | STN volume (mm^3^)-Right Hemisphere | | STN volume (mm^3^)-Left Hemisphere | | STN Mean Susceptibility (ppb)-Right Hemisphere | | STN Mean Susceptibility (ppb)-Left Hemisphere | |
| --- | --- | --- | --- | --- | --- | --- | --- | --- |
|  | Mean | Standard Deviation | Mean | Standard Deviation | Mean | Standard Deviation | Mean | Standard Deviation |
| subject 1 | 81 | 14.0 | 80 | 11.6 | 97 | 17.9 | 130 | 29.5 |
| subject 2 | 47 | 9.6 | 17 | 10.0 | 72 | 6.5 | 62 | 5.2 |
| subject 3 | 49 | 10.0 | 41 | 14.0 | 88 | 4.9 | 82 | 17.0 |
| subject 4 | 40 | 13.3 | 41 | 8.3 | 105 | 14.6 | 105 | 10.0 |
| Total | Average mean | Standard error of the mean | Average mean | Standard error of the mean | Average mean | Standard error of the mean | Average mean | Standard error of the mean |
|  | 54 | 9.1 | 45 | 13.1 | 91 | 7.20 | 95 | 14.8 |

**Supplementary Table 4.** STN volume and mean susceptibility values for four volunteers who were scanned 5 times each at four different sites.


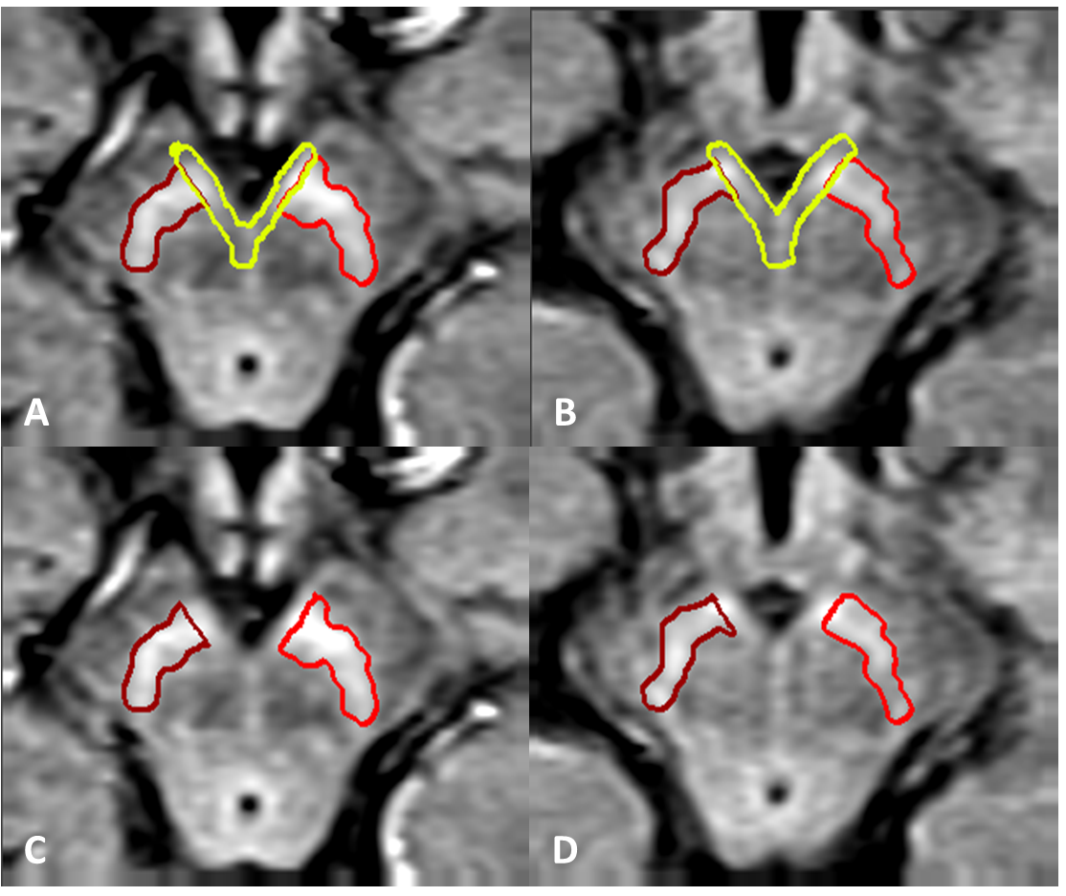


**Supplementary Figure 1.** Four consecutive midbrain NM images. (A,B) The yellow boundary is the VTA structure. (C,D) The VTA is removed from the NM-rich region shown in the corresponding slices.

**
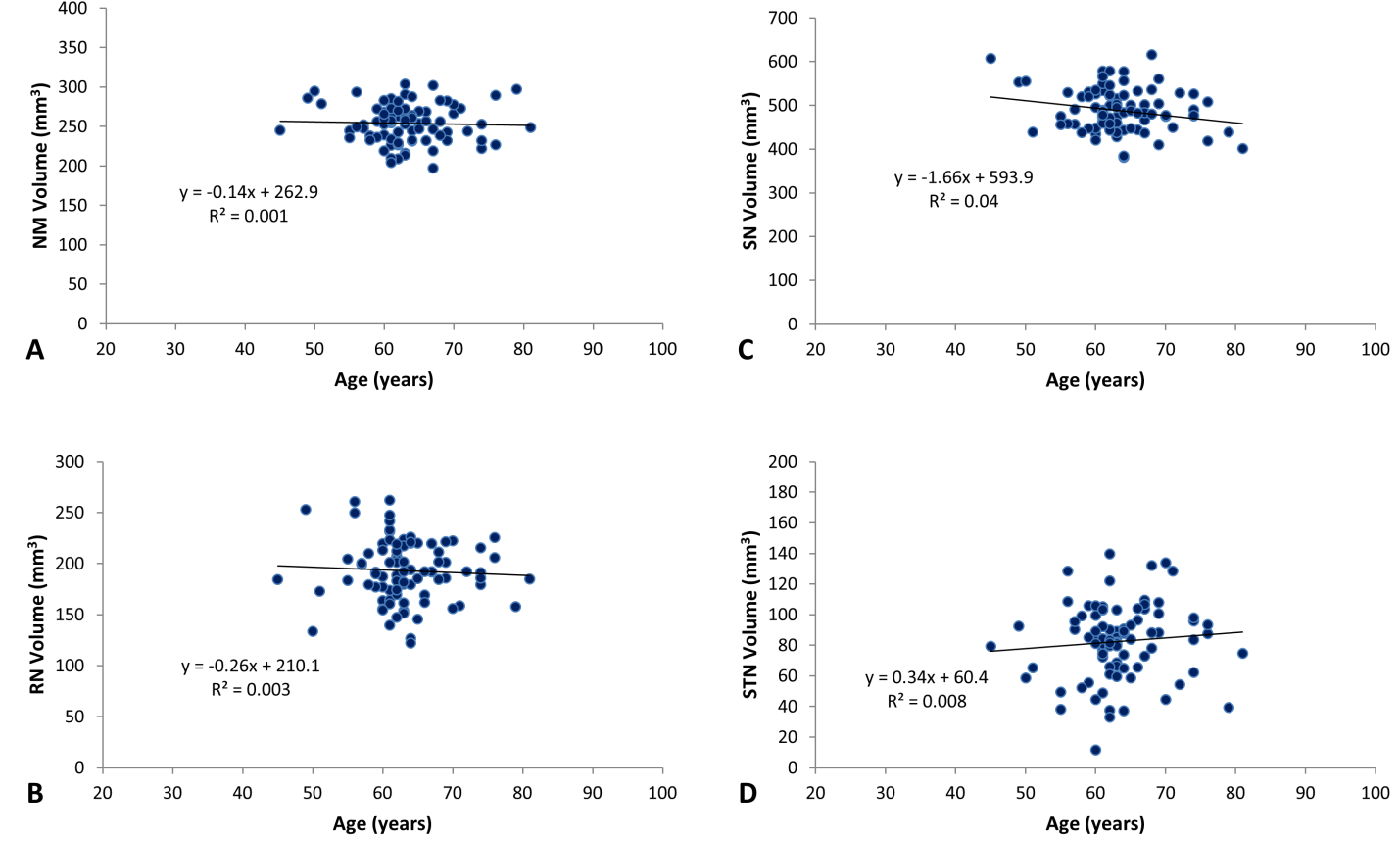
**

**Supplementary Figure 2.** Volume of the NM-rich region, RN, SN and STN as a function of age associated with the merged dataset (87 healthy controls). The p-values associated with each correlation are 0.30, 0.61, 0.05 and 0.40, respectively.

**
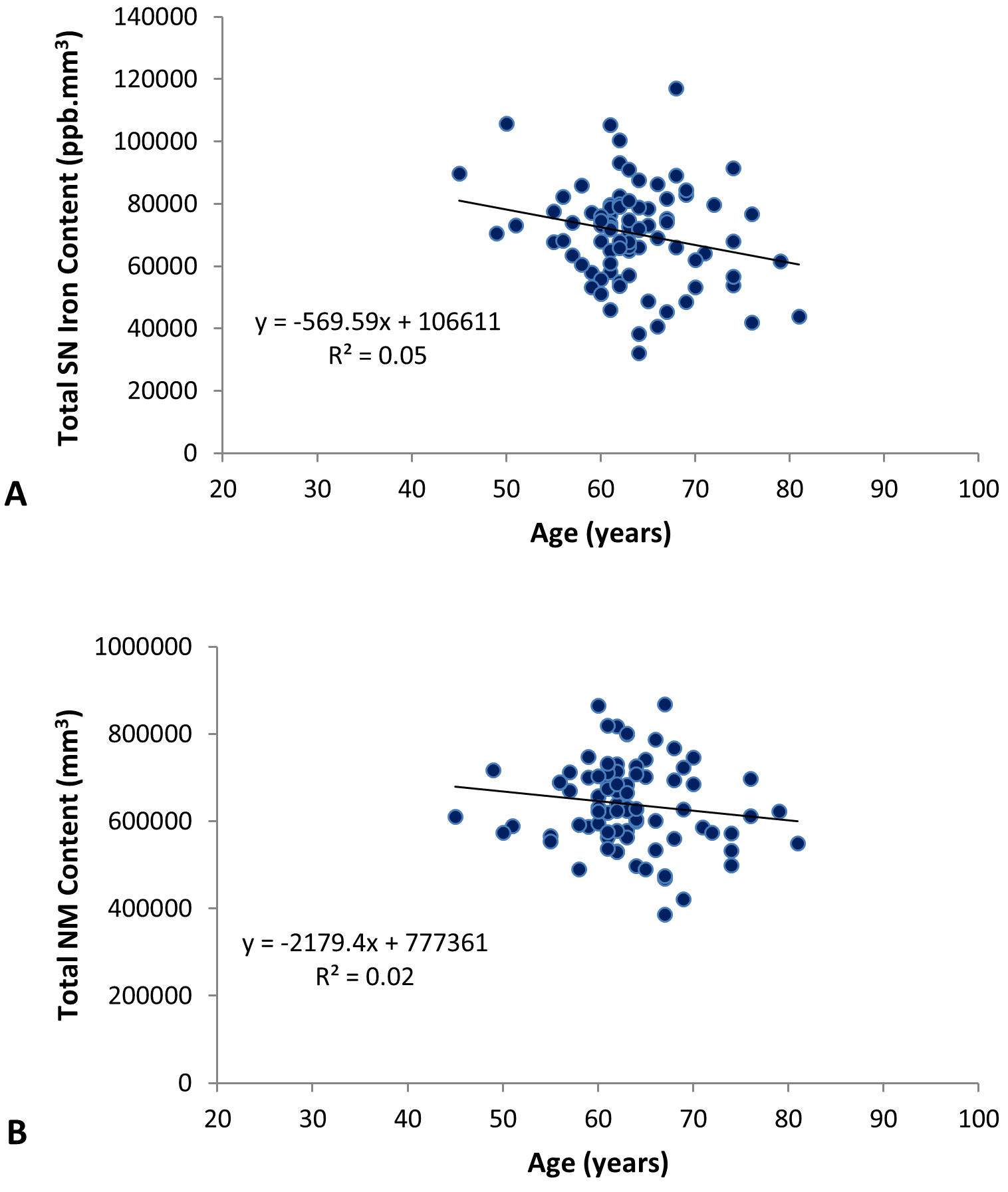
**

**Supplementary Figure 3.** A) Total iron content of the SN (p = 0.02), and B) total NM content (p = 0.21) as a function of age for the merged dataset (87 healthy controls).

**
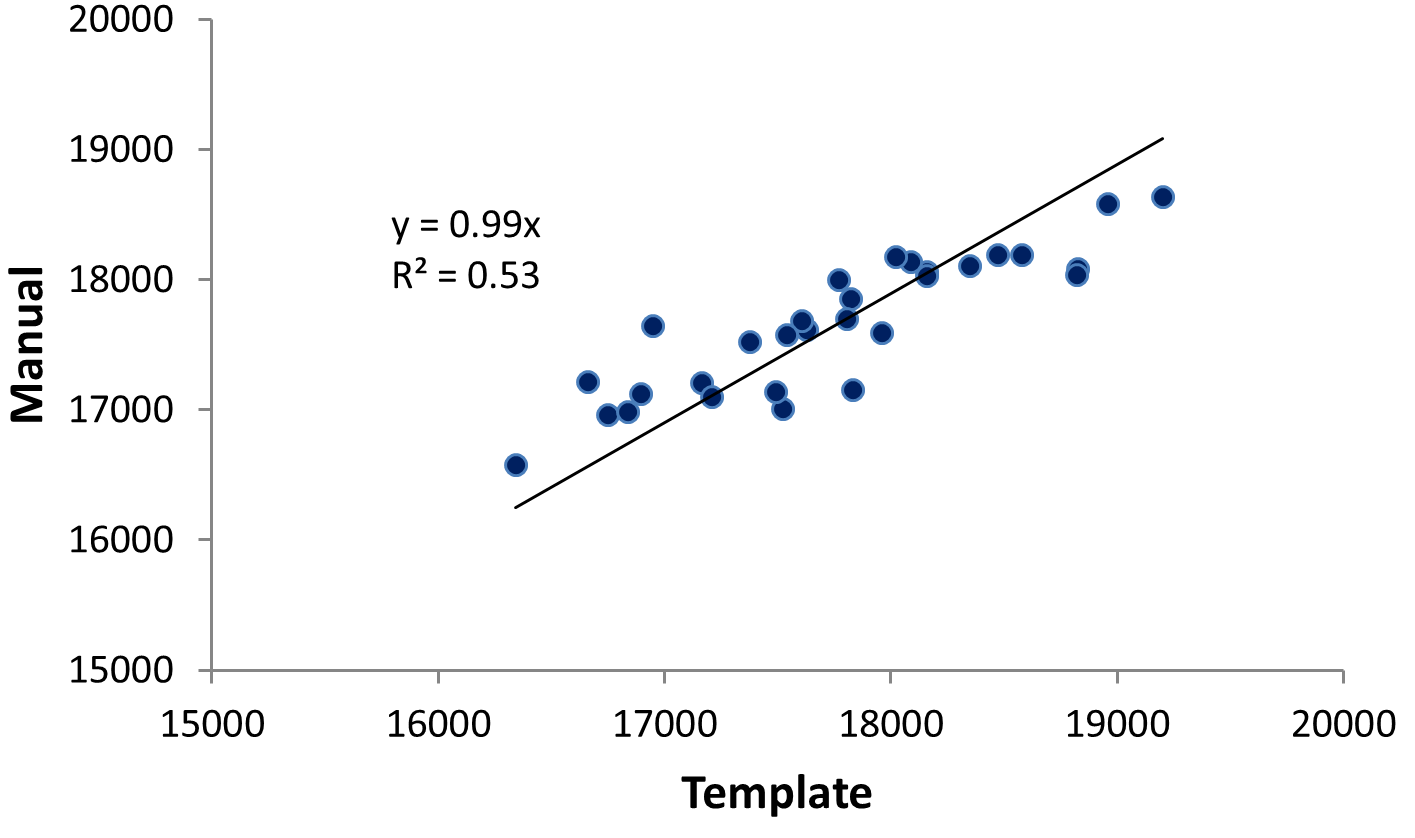
**

**Supplementary Figure 4.** Agreement between the NM background manual and template measurements for 30 healthy controls. NM measures are in arbitrary units.

**
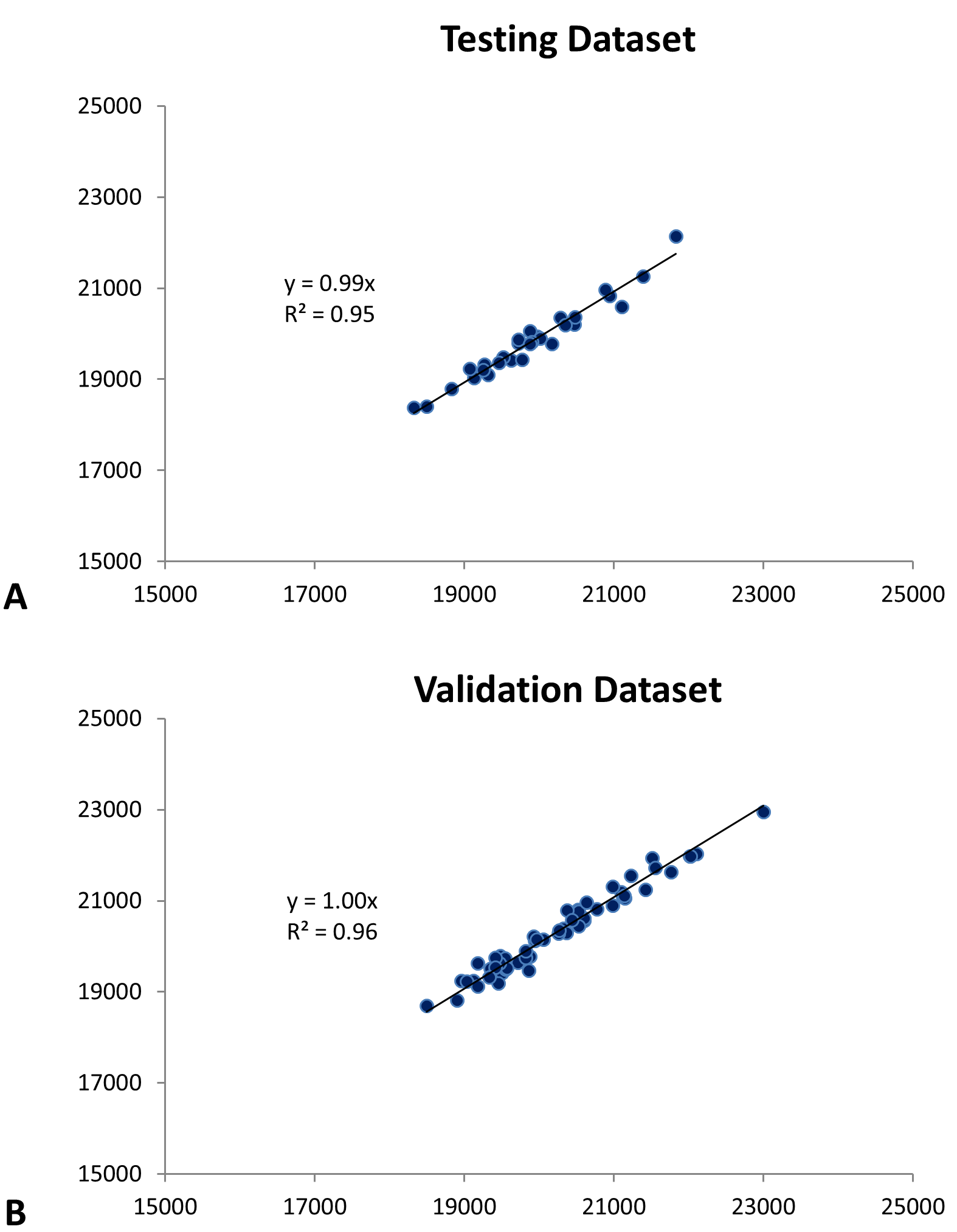
**

**Supplementary Figure 5.** Correlation between the NM mean intensity measurements resulting from the manual and template segmentations for the A) 30 test cases, and B) 57 validation cases. (p-value < 0.001 for both plots).


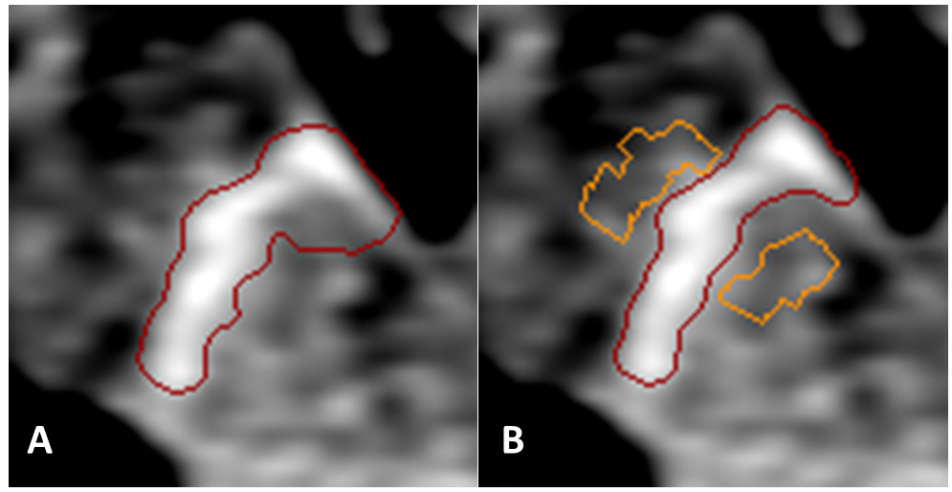


**Supplementary Figure 6.** A) NM boundary before DPA, B) Background boundaries shown relative to the NM boundary after DPA. The orange boundary is the background region after transformation from the template space to the original space. Using the background value plus 1000 makes it easier to refine the DPA boundary to provide a faster convergence and to use a smaller and safer search radius to prevent the algorithm from leaking outside the original boundary.

.


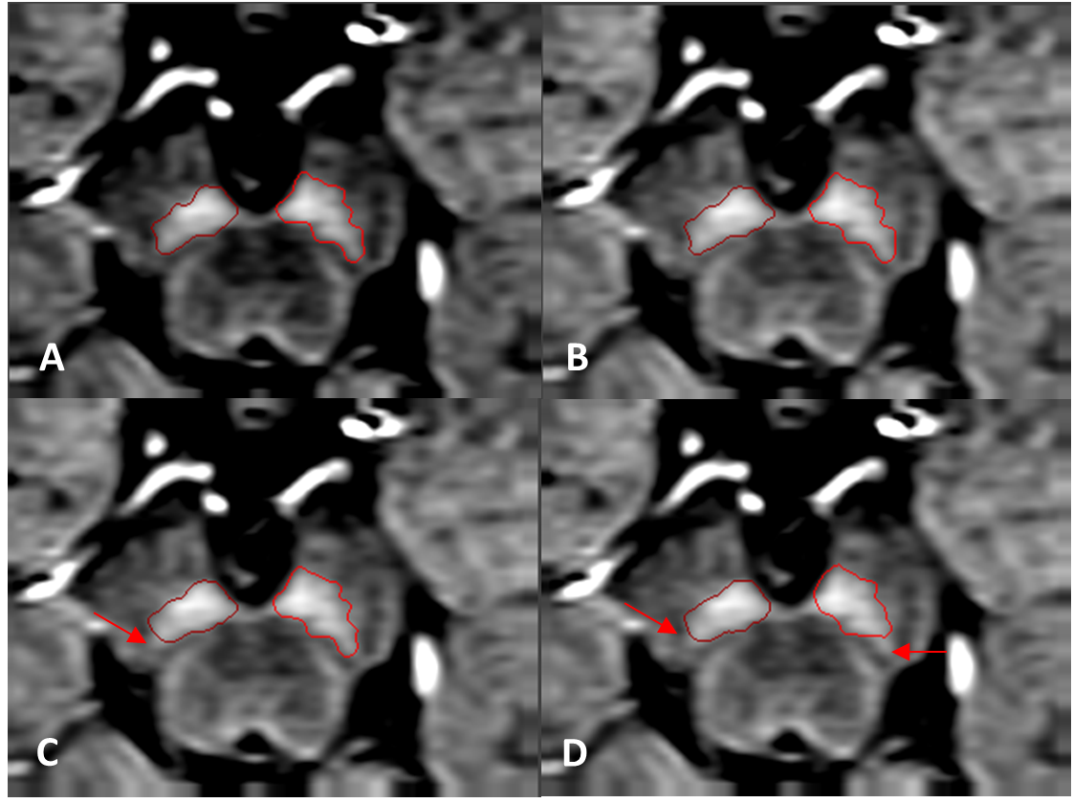


**Supplementary Figure 7.** The choice of α can dramatically affect the final DPA boundary.

A) α = 0.05; B) α = 0.10; C) α = 0.15; and D) α = 0.20. Note that lower values of α lead to less smoothing from the radius constraint, while larger values of α lead to more smoothing.


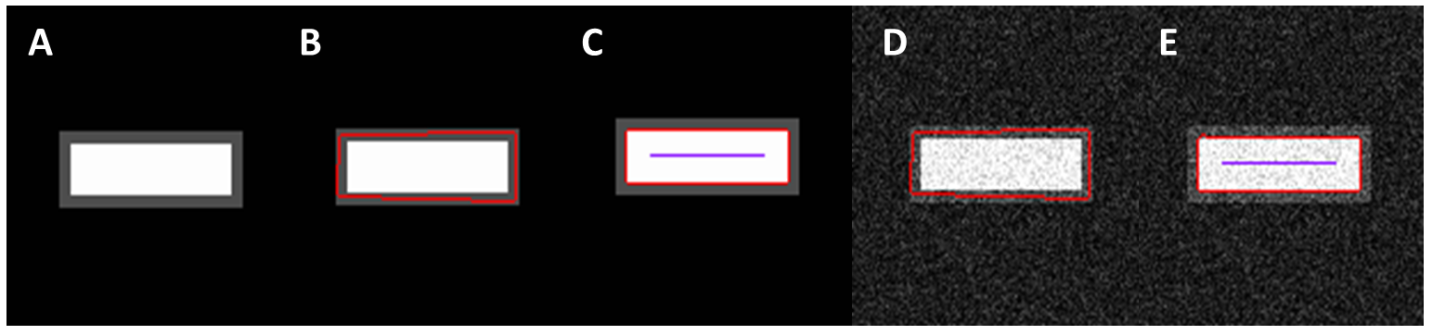


**Supplementary Figure 8.** A) No noise case: the model image shows a rectangular shape with two different intensities. B) A region was drawn around the central area, C) and then the centerline was found and updated after applying the DPA for 5 iterations. D) With noise: the same drawing was then used in the presence of a CNR of 7:1 based on the difference in the signal intensities of the central region and the framed region within the second rectangular boundary. E) After 5 iterations, the correct boundary was found providing the correct area of 1400 pixels.


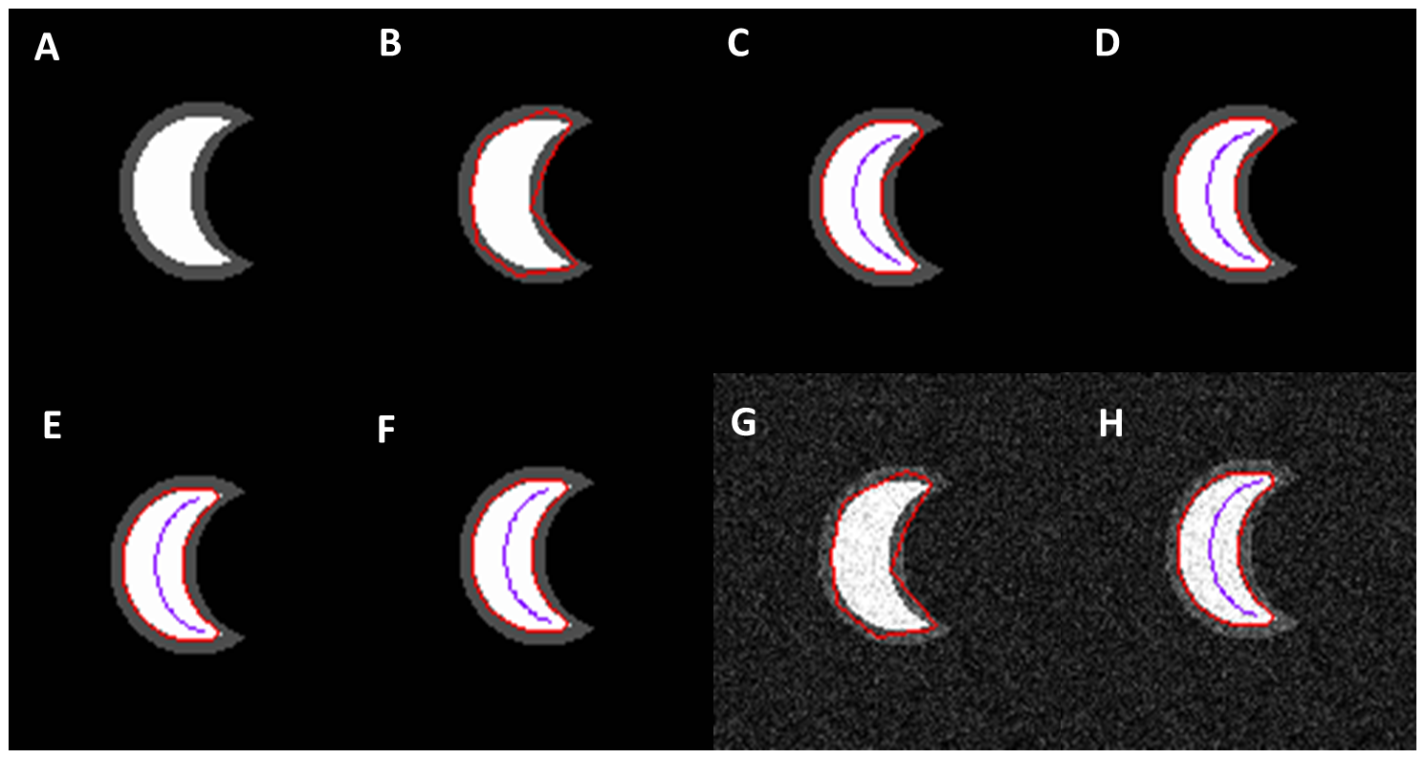


**Supplementary Figure 9.** A) No noise case: the model image shows the moon shape with two different intensities. B) A region was drawn around the central area, C) and then the centerline was found and updated after each of the DPA for 5 iterations. D) First 10 iterations, E) then 15 iterations, F) and then with just 5 iterations using the adaptive Otsu threshold approach were evaluated. G) With noise: The same drawing was then used in the presence of a CNR of 7:1 based on the difference in the signal intensities of the central region and the framed region within the second rectangular boundary, H) and again using just 5 iterations using the adaptive Otsu threshold approach.


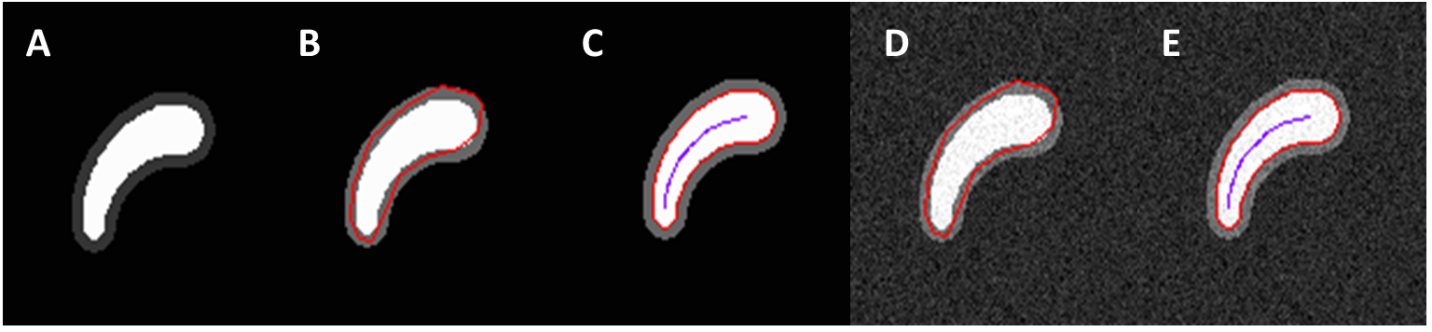


**Supplementary Figure 10.** A) No noise case: the model image shows the cashew shape with two different intensities. B) A region was drawn around the central area, (C) and then the centerline was found and updated after DPA 5 iterations. D) With noise: The same drawing was then used in the presence of a CNR of 7:1 based on the difference in the signal intensities of the central region and the framed region within the second rectangular boundary. E) The final result.

*
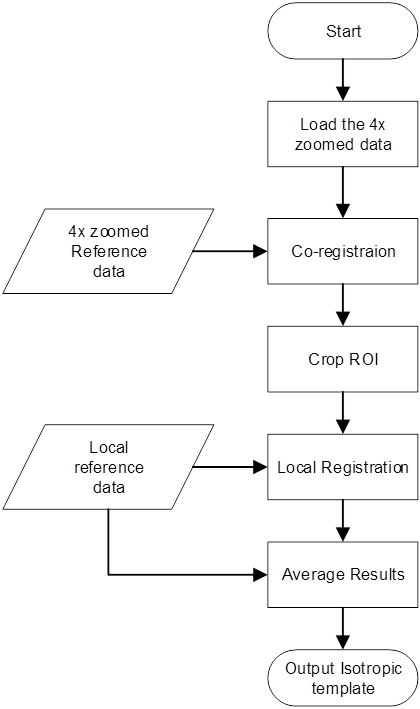
*

**Supplementary Figure 11.** Flowchart of the automated segmentation algorithm.

**References**

Jiang, J., Haacke, E. M., & Dong, M. (2007). Dependence of vessel area accuracy and precision as a function of MR imaging parameters and boundary detection algorithm. *J Magn Reson Imaging, 25*(6), 1226-1234. doi:10.1002/jmri.20918

Liu, Y., Li, J., He, N., Chen, Y., Jin, Z., Yan, F., & Haacke, E. M. (2020). Optimizing neuromelanin contrast in the substantia nigra and locus coeruleus using a magnetization transfer contrast prepared 3D gradient recalled echo sequence. *Neuroimage, 218*, 116935. doi:10.1016/j.neuroimage.2020.116935

Otsu, N. (1979). A threshold selection method from gray-level histograms. *IEEE T Syst Man Cy B, 9*, 62-66. doi:10.1109/TSMC.1979.4310076

Zhang, T. Y., & Suen, C. Y. (1984). A fast parallel algorithm for thinning digital patterns. *Communications of the ACM, 27*(3), 236-239. doi:10.1145/357994.358023
